# Hybrid Chemo-, Bio-, and Electrocatalysis for Atom-Efficient Deuteration of Cofactors in Heavy Water

**DOI:** 10.1021/acscatal.0c03437

**Published:** 2021-02-11

**Authors:** Jack S. Rowbotham, Holly A. Reeve, Kylie A. Vincent

**Affiliations:** Department of Chemistry, Inorganic Chemistry Laboratory, University of Oxford, South Parks Road, Oxford OX1 3QR, United Kingdom

**Keywords:** Chemoenzymatic (Chemo-Bio), Electroenzymatic, Heterogenous biocatalysis, Site-separated catalysis, Isotope labeling, Isotopic selectivity, ^2^H_2_O (D_2_O), Dihydrogen gas (H_2_)

## Abstract

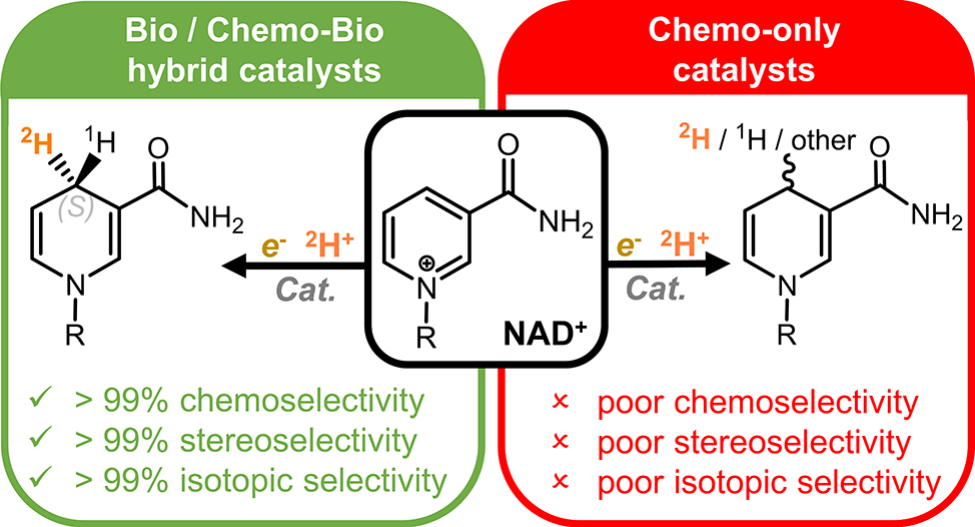

Deuterium-labeled nicotinamide cofactors such as [4-^2^H]-NADH can be used as mechanistic probes in biological redox processes and offer a route to the synthesis of selectively [^2^H] labeled chemicals *via* biocatalytic reductive deuteration. Atom-efficient routes to the formation and recycling of [4-^2^H]-NADH are therefore highly desirable but require careful design in order to alleviate the requirement for [^2^H]-labeled reducing agents. In this work, we explore a suite of electrode or hydrogen gas driven catalyst systems for the generation of [4-^2^H]-NADH and consider their use for driving reductive deuteration reactions. Catalysts are evaluated for their chemoselectivity, stereoselectivity, and isotopic selectivity, and it is shown that inclusion of an electronically coupled NAD^+^-reducing enzyme delivers considerable advantages over purely metal based systems, yielding exclusively [4*S*-^2^H]-NADH. We further demonstrate the applicability of these types of [4*S*-^2^H]-NADH recycling systems for driving reductive deuteration reactions, regardless of the facioselectivity of the coupled enzyme.

## Introduction

**Scheme 1:**
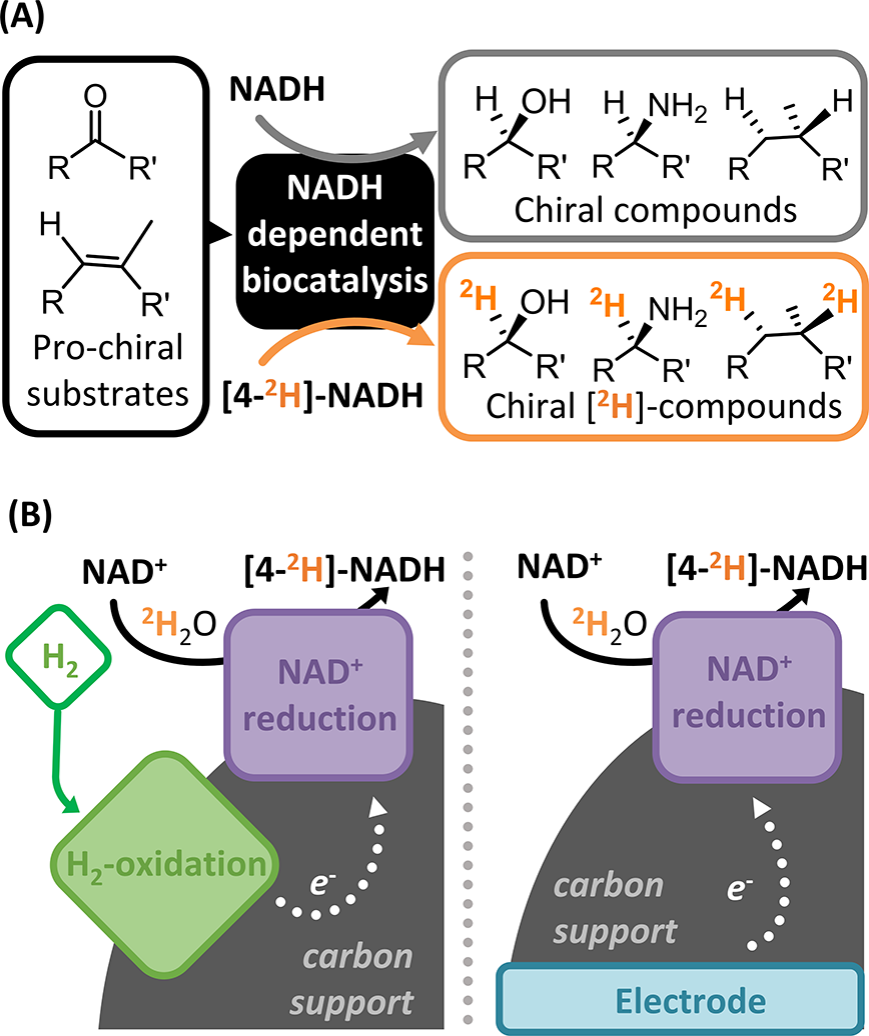
Biocatalytic Deuteration via Formation of the Deuterium-Labeled Cofactor [4-^2^H]-NADH: (A) [4-^2^H]-NADH May Be Used to Prepare Specifically [^2^H] Labeled Organic Products by Enzymatic Reductive Deuteration; (B) Atom-Efficient Routes to NAD^+^ Reduction (Driven by H_2_ Gas or an Electrode) Can Be Used in ^2^H_2_O to Generate [4-^2^H]-NADH For complete structures of the cofactors see Figure S1 in the Supporting Information.

Reduced nicotinamide cofactors (NADH and NADPH) are used as electron carriers in many biological reactions and are critical to respiration and CO_2_ fixation, as well as in the biosynthesis of complex molecules. Consequently, isotopologues of these cofactors, and in particular the deuterated molecule [4-^2^H]-NADH, have long been used to probe the kinetics and mechanisms of a multitude of enzyme-driven reactions.^1−3^ From a synthetic standpoint, deuterated cofactors also represent an opportunity for labeled chemical production, where a vast array of commercially available NADH-dependent C=O-, C=C-, and C=N-bond reductases may be employed for deuterium transfer from [4-^2^H]-NADH to a target molecule (Scheme 1A). As such, integrating a suitable [4-^2^H]-NADH recycling system into biocatalytic reactions offers a versatile pathway to α-deuterium-labeled chiral molecules for analytical and pharmaceutical applications, notably in the synthesis of heavy-drug analogues.^4,5^ Research into new routes to [^2^H] compounds has intensified recently, following the clinical approval of the first deuterodrug (*deutetrabenazine*),^6^ with other candidates in the pipeline.^7−10^

Within the wider field of redox biocatalysis, there have been concerted efforts to transition from established forms of biocatalytic cofactor recycling (based on organic reductants such as formate and glucose) to alternative, atom-efficient methods (based on H_2_, photochemical, and electrochemical reduction).^11−21^ Simultaneously, in the field of deuterium labeling, there has been a marked effort to design catalysts that utilize heavy water (^2^H_2_O) as the source of the isotopes, for reasons of cost, safety, and sustainability.^22−31^ In line with these developments, we have recently reported on enzymatic methods that deliver [4-^2^H]-NADH recycling using H_2_ plus ^2^H_2_O.^4^ These approaches employ a NiFe hydrogenase unit to oxidatively cleave H_2_ and transfer electrons to a separate flavin-dependent NAD^+^ reductase unit, which converts NAD^+^ to [4-^2^H]-NADH with a deuteron (^2^H^+^) from the solution (Scheme 1B). The hydrogenase and NAD^+^ reductase can be electronically linked as part of a single enzyme or as distinct moieties immobilized on a conductive carbon support.^4,32^ The separation of the two catalytic sites enables a ^2^H-labeled product ([4-^2^H]-NADH) to be formed in the presence of an unlabeled reductant (H_2_). In the work presented here, we seek to further establish this site-separated approach by comparing electrocatalytic, chemocatalytic, and biohybrid systems.

The development of multifunctional “chemo-bio” catalysts represents not only a considerable opportunity but also a notable challenge.^33,34^ Many recent examples of combined metal/enzyme systems have demonstrated the power to perform challenging reactions more easily but also draw attention to potential problems, such as the need for compromise conditions and the risk of mutual inactivation of the two catalysts.^35−38^

**Figure 1:**
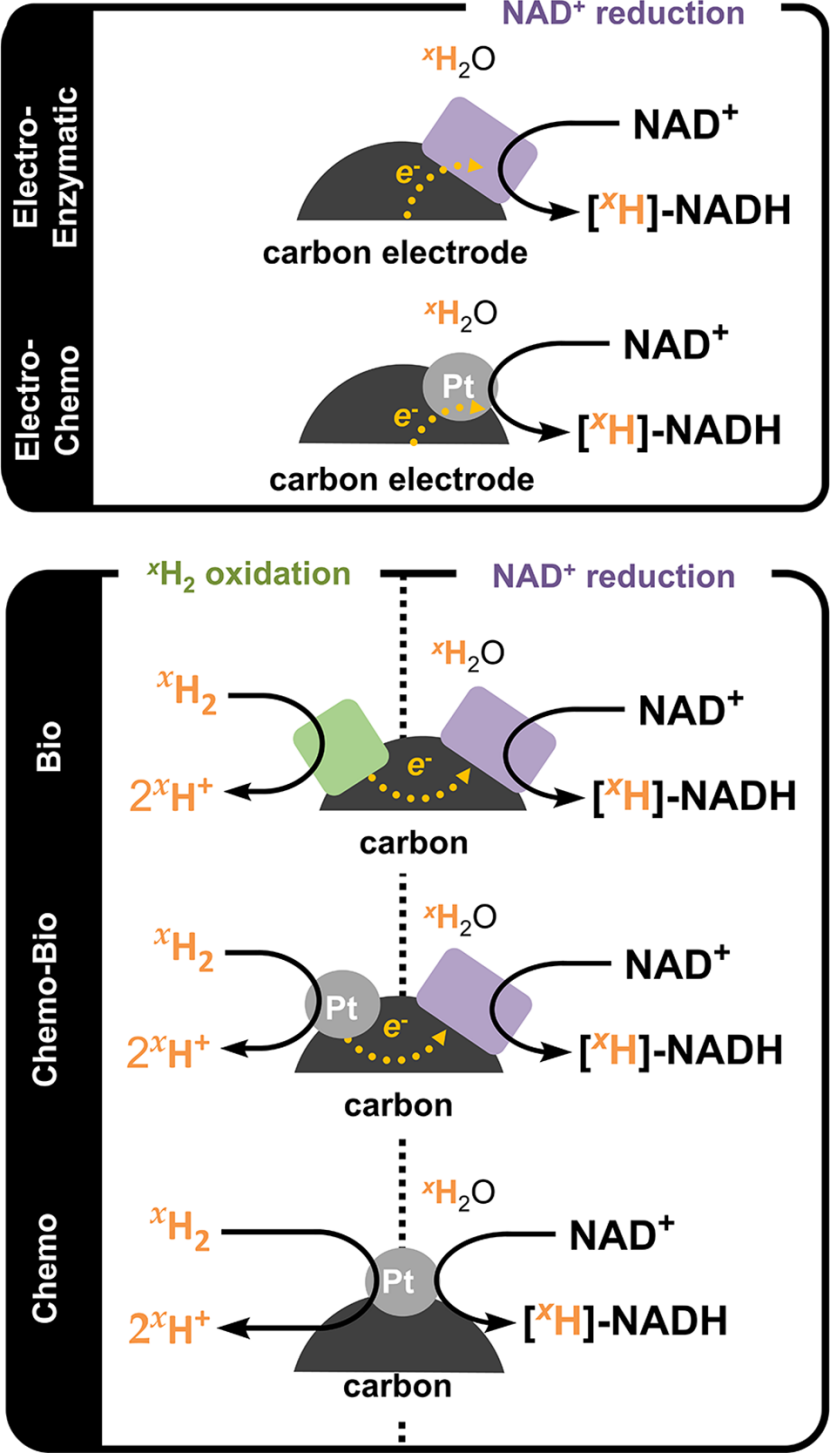
The suite of heterogeneous catalysts investigated here for electrode or ^*x*^H_2_-driven generation of the labeled cofactor [4-^2^H]-NADH. For the electrode-driven approaches (top), either *Electro-Enzymatic* (NAD^+^ reductase on carbon on electrode) or *Electro-Chemo* (Pt/C on electrode) systems were used. For the ^*x*^H_2_-driven methods (bottom), each catalyst comprises a site for H_2_ oxidation and a site for NAD^+^ reduction. The *Bio* system combines a hydrogenase (green square) and NAD^+^ reductase (purple rectangle), the *Chemo-Bio* system combines Pt (gray circle) and a NAD^+^ reductase, and the *Chemo* system utilizes just Pt for both half-reactions (all immobilized on a conductive carbon support).

The full suite of NAD^+^-reducing catalysts explored in this study is shown in Figure 1. Electrocatalysts were prepared by coating a graphite electrode with either Pt/C (*Electro-Chemo* system) or an NAD^+^-reductase enzyme immobilized on carbon (*Electro-Enzymatic* system). For H_2_-driven NAD^+^ reduction, three catalyst systems were studied: the previously described *Biocatalytic* system (NiFe hydrogenase and NAD^+^ reductase),^4^ a *Chemo-Biocatalytic* system (Pt/C and NAD^+^ reductase), and an entirely *Chemocatalytic* system (Pt/C). These catalysts feature sites for H_2_ oxidation (Pt or enzyme) and NAD^+^ reduction (Pt or enzyme) connected through their coimmobilization on the electronically conducting carbon support. All of the catalysts discussed in the paper were straightforward to prepare, and their heterogeneous nature simplified their handling and separation from the chemical products.

In an evaluation of the catalyst systems, their chemoselectivity toward the production of the biologically active form of the cofactor (1,4-NADH, hereafter referred to as NADH for simplicity) is an essential criterion. Electrocatalysts and metal-based chemocatalysts have often been found to generate biologically inactive side products (such as NAD_2_ dimers and 1,6- or 1,2-NADH), giving rise to rapid depreciation of the cofactor in high-turnover applications.^14,18−20,39−41^

Additional considerations in assessing the performance of the catalysts are the isotopic purity of the product (the % ^2^H of the [4-^2^H]-NADH relative to the ^2^H_2_O solvent) and the corresponding stereochemical purity (the ratio of [4*S*-^2^H]-NADH to [4*R*-^2^H]-NADH, dependent on which face of the nicotinamide ring the ^2^H atom is added to).^42^ An evaluation of all of these factors enables a discussion of the suitability of the various catalysts for coupling to different NADH-dependent enzymes for the preparation of valuable selectively deuterated molecules.

## Catalyst Preparation

In this work, five catalytic systems were explored for generating deuterated NADH, as defined in Figure 1. The catalysts were all prepared utilizing enzymes expressed in their native bacterial hosts, alongside widely available commercial carbon black and Pt/C materials (see Section 1.1.3 in the Supporting Information for full details).

The hydrogenase 1 from *Escherichia coli* was selected for its O_2_ tolerance and relative ease of preparation. It has a NiFe active site capable of H_2_ oxidation at low overpotentials and a chain of FeS clusters for electron transfer.^20^ The NAD^+^ reductase candidate is a flavoenzyme from *Ralstonia eutropha* (also known as *Cupriavidus necator*) capable of bidirectional NAD^+^ reduction and, as with the hydrogenase, contains a chain of FeS clusters for electron transfer.^43^

Two commercial carbon blacks were used in this study as catalyst supports: BP2000 (from Cabot Corp.) and 20 wt % Pt/C (HiSPEC 3000 from Alfa Aesar). Both have previously been characterized extensively by us (BP2000)^44^ and other authors (HiSPEC 3000 Pt/C).^45−48^ These supports were selected primarily for their electron transfer properties and ease of handling, though we have previously demonstrated that a wide range of carbon materials is suitable for this type of heterogeneous biocatalysis.^44,49^ Enzymes were immobilized (or coimmobilized) onto the carbon black supports by a simple adsorption process. Given the lack of covalent attachment, the adsorption leads to surprisingly robust catalysts, and the systems show minimal enzyme leaching, even when they are operated under flow conditions.^32^

Electrocatalyst systems were prepared on a pyrolytic graphite edge (PGE) rotating-disk electrode (RDE). The PGE was coated with the carbon black and NAD^+^ reductase enzyme or Pt/C. While it is possible to immobilize the NAD^+^ reductase directly onto the PGE electrode, the additional carbon support gives rise to a greatly increased surface area and hence enables a higher effective catalyst loading to be achieved.

In summary (and following the ordering in Figure 1), the formulations of the catalyst systems were as follows: (i) *Electro-Enzymatic*, NAD^+^ reductase on carbon black (BP2000) on PGE; (ii) *Electro-Chemo*, 20 wt % Pt/C (HiSPEC 3000) on PGE; (iii) *Bio*, hydrogenase and NAD^+^ reductase coimmobilized on carbon black (BP2000); (iv) *Chemo-Bio*, NAD^+^ reductase on 20 wt % Pt/C (HiSPEC 3000); (v) *Chemo*, 20 wt % Pt/C (HiSPEC 3000). Note that the enzyme/metal ratio in the *Chemo-Bio* system was found to be a key variable for determining the selectivity and is explored in detail below.

## Results and Discussion

Each of the catalyst systems defined in Figure 1 was first investigated for the generation of [^2^H]-labeled NADH when it was supplied with NAD^+^ in ^2^H_2_O and electrons either from an electrode or from the oxidation of H_2_.

In the case of the *Electro-Chemo* and *Electro-Enzymatic* electrode-driven systems, a standard bulk electrolysis setup was utilized (described in Section S.1.2.1 in the Supporting Information). Here, the PGE working electrode was coated with the various catalysts and immersed in a stirred buffered solution of NAD^+^ alongside a reference electrode (saturated calomel electrode (SCE), with potentials then corrected to the standard hydrogen electrode (SHE)) and a counter electrode. The counter electrode was separated from the bulk solution by means of a molecular sieve frit, which prevents additional reactions of the cofactor at this site. A potentiostat was used to control the potential, and the current through the working electrode could then be measured. The current resulting from a flow of electrons from the electrode to a substrate is referred to as the “catalytic current” and can be used to gauge the activity of the catalysts. By poising the electrode at a suitable potential over a period of time, NADH can be prepared at sufficient levels for analysis. In this case, the selected value was −560 mV vs SHE, which represented a mild overpotential with respect to the NAD^+^/NADH couple,^43^ thereby minimizing the voltage input and maximizing energy efficiency. When the unmodified pyrolytic graphite edge electrode was held at this potential, no catalytic current was observed, indicating that no reaction had taken place (Figure S4 in the Supporting Information). In contrast, a negative current consistent with electrocatalytic NAD^+^ reduction was observed at the same potential for the NAD^+^ reductase modified *Electro-Enzymatic* catalyst, and NADH formation was observed (see later discussion). A negative current was also observed with the *Electro-Chemo* system at −560 mV; however, a UV analysis demonstrated that this was not due to NAD^+^ reduction and therefore likely arose from deuteron reduction. Stepping the potential to more negative values did give rise to currents corresponding to NAD^+^ reduction for the *Electro-Chemo* system and the bare electrode, though yellowing of the solution was indicative of the formation of biologically inactive side products of the cofactor (see Section S.2.1 in the Supporting Information).^18,40,41^

Alternatively to the electrode-driven systems, H_2_ was also investigated as a reductant. Catalytic hydrogenations were carried out in ^2^H_2_O and under a H_2_ atmosphere (2 bar), with the products being analyzed in a manner similar to the electrochemical experiments. For all three H_2_-driven systems (*Bio*, *Chemo-Bio*, and *Chemo*) a reaction with NAD^+^ was found to occur under the mild (low temperature and pressure) conditions used.

**Figure 2:**
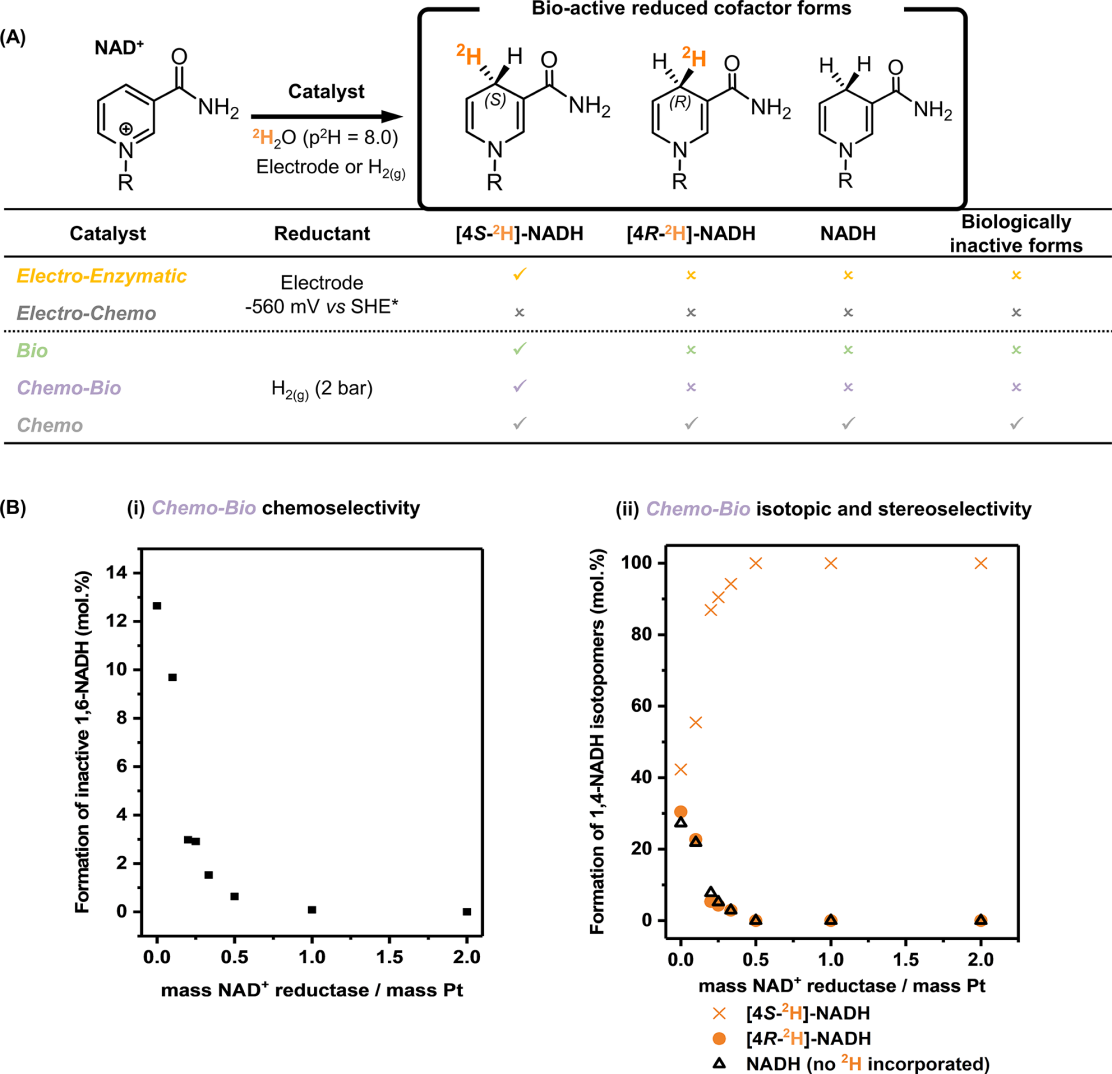
Selectivity of electrode and H_2_-driven catalyst systems for generation of [^2^H]-labeled NADH. (A) Distribution of products formed by each of the *Electro-Enzymatic*, *Electro-Chemo*, *Bio*, *Chemo-Bio*, and *Chemo-* catalysts, studied under comparable conditions. The three catalysts containing an NAD^+^ reductase unit were found to be selective for a single product: [4*S*-^2^H]-NADH. Reaction mixtures contained 4 mM NAD^+^ in ^2^H_2_O (p^2^H 8.0), and the products were analyzed using HPLC and ^1^H NMR (Figures S5 and S6 in the Supporting Information). For electrode-driven experiments, the electrode was poised at −560 mV vs SHE. For H_2_-driven reactions the catalysts were operated in H_2_-saturated solution (2 bar of H_2_). (B) Experiments to determine the relationship between the enzyme/metal mass ratio and the chemoselectivity, isotopic selectivity, and stereoselectivity of the *Chemo-Bio* catalyst for making [4*S*-^2^H]-NADH from NAD^+^ under ^1^H_2_ gas in ^2^H_2_O. Comparisons of selectivity were all made at similar levels of conversion (90–95%). (i) Chemoselectivity (as measured by HPLC): in converting NAD^+^ to 1,4-NADH, the formation of a biologically inactive side product (1,6-NADH) by Pt/C catalysts was increasingly suppressed by the addition of NAD^+^ reductase. (ii) Isotopic selectivity and stereoselectivity (as measured by ^1^H NMR): of the 1,4-NADH formed, a distribution of isotopomers was observed consisting of [4*R*-^2^H]-NADH, [4*S*-^2^H]-NADH, and unlabeled (no ^2^H) NADH. Again, increasing the NAD^+^ reductase loading on the Pt/C catalyst led to the formation of exclusively [4*S*-^2^H]-NADH. Note: (*) The *Electro-Chemo* system was also operated at more negative potentials, giving rise to NADH and biologically inactive forms (see Section S.2.1 in the Supporting Information).

In analyzing the products of NAD^+^ reduction, Wang and co-workers have highlighted the importance of employing a number of complementary techniques.^14,39^ Here we used UV–vis spectroscopy and HPLC to establish the formation of NADH, combined with ^1^H NMR spectroscopy to confirm the stereotopic and isotopic purity of the products (see Section S.1.2 in the Supporting Information). The results of these analyses are summarized in Figure 2. It was found that all catalysts incorporating the NAD^+^ reductase enzyme (the *Electro-Enzymatic*, *Bio-,* and *Chemo-Bio* systems) generated only NADH deuterated at the [4]-position on the nicotinamide ring: [4-^2^H]-NADH. Addition of a single ^2^H atom at the [4]-position leads to the formation of an isotopically induced stereocenter (see Figure 2A) and therefore the possibility of forming either the *R* or *S* isomer of [4-^2^H]-NADH. In addition to showing full regioselectivity, the *Electro*-*Enzymatic*, *Bio-,* and *Chemo-Bio* systems produced only the *S* isomer, [4*S*-^2^H]-NADH. In contrast, the Pt-based *Chemo* system gave no stereochemical control (generating a mixture of [4*R*-^2^H]- and [4*S*-^2^H]-NADH) and also led to the production of nonlabeled NADH, as well as a biologically inactive cofactor (see Figure S6 in the Supporting Information).^14,40,50,51^

It is significant that the product regio- and stereoselectivity for the *Chemo-Bio* system exactly matches that for the *Bio* system, as this suggests that the NAD^+^ reduction half-reaction of the former occurs solely at the NAD^+^ reductase, avoiding nonselective NAD^+^ reduction at Pt. In order to explore this effect further, different loadings of NAD^+^ reductase on the Pt/C were trialed under the same conditions. Here it was found that, at low loadings of NAD^+^ reductase (<0.5 mass ratio relative to Pt), conversion proceeded to >90% but up to 13% of the product was 1,6-NADH instead of the desired 1,4-NADH (the presence of a yellow hue was also evidence of other trace impurities below the level of quantification). As the mass of the enzyme was increased relative to the amount of Pt, the conversion remained high (>90%), the formation of 1,6-NADH was suppressed in favor of 1,4-NADH (as shown in Figure 2B(i); see full data in Figure S6 in the Supporting Information), and the yellow hue disappeared. Analysis of the 1,4-NADH generated in the same reactions revealed a mixture of [4*S*-^2^H], [4*R*-^2^H], and nondeuterated isotopomers until the 0.5 enzyme/metal mass ratio was surpassed (see Figure 2B(ii)). For catalysts with an enzyme/metal mass ratio of greater than 0.5, [4*S*-^2^H]-NADH was the sole product, confirming that the NAD^+^ reductase was the only site of NAD^+^ reduction when it was present in sufficiently high loadings. Given the low selectivity of the hybrid enzyme/metal system at low enzyme loadings, the term *Chemo-Bio* catalyst is therefore used generally to refer to the catalyst with an enzyme/metal mass ratio of 1.0. Aside from selectivity, it is interesting to note that addition of the NAD^+^ reductase enzyme to the Pt/C system to create the *Chemo*-*Bio* system also appeared to have a beneficial effect on the activity of the catalyst in the deuteration reactions (Figure S7 in the Supporting Information).

For completeness, it was also confirmed in a control experiment that the NAD^+^ reductase alone on carbon is unable to extract electrons from H_2_; thus, the Pt in the *Chemo-Bio* system must be solely responsible for H_2_ oxidation. The contrast between the mechanisms of the *Chemo* and *Chemo-Bio* catalysts is then clear: in the *Chemo* system, the Pt is responsible for both H_2_ oxidation and NAD^+^ reduction, and in the *Chemo*-*Bio* system the Pt is responsible for just H_2_ oxidation, with the NAD^+^ reduction occurring only through the NAD^+^ reductase enzyme.

The stereocontrol afforded by the *Bio*, *Chemo-Bio*, and *Electro-Enzymatic* systems may consequently be attributed to the NAD^+^ reductase moiety. Here, the enzyme transfers a deuteride unit (^2^H^–^) selectively to the *si* face of the nicotinamide ring of the oxidized cofactor, giving rise to [4*S*-^2^H]-NADH. This is consistent with the crystallographic structure of the highly conserved Rossmann fold of the NAD^+^ reductase moiety, in which a homology model to the cofactor-bound form of complex I shows the *si* face of the cofactor docked adjacent to the flavin mononucleotide catalytic site (Figure S8 in the Supporting Information).^52,53^ Depreciation of the stereopurity of the product is therefore a consequence of nonselective NAD^+^ reduction occurring at Pt/C sites, not within the active site of the NAD^+^ reductase enzyme.

**Figure 3:**
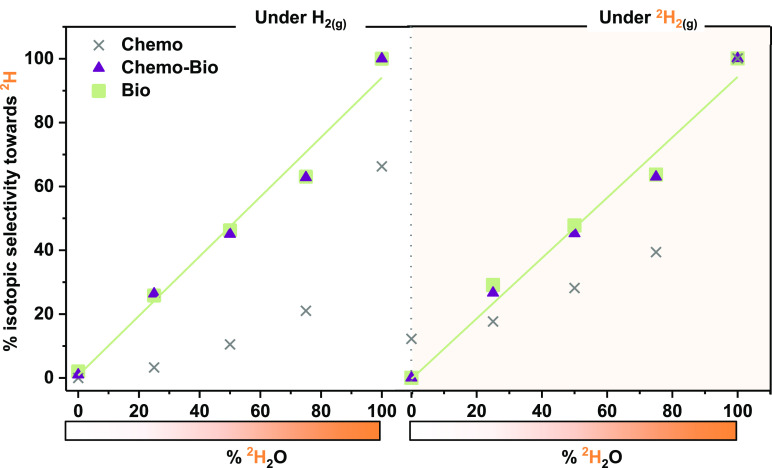
The isotopic selectivity of H_2_-driven NADH generation from NAD^**+**^ by *Bio*, *Chemo-Bio*, and *Chemo* catalysts in the presence of ^*x*^H_2_O and ^*x*^H_2_. Analogous experiments were performed in varying mixtures of ^1^H_2_O and ^2^H_2_O, under either (left) ^1^H_2_ or (right) ^2^H_2_ gas. Lines of best fit through data for the *Bio* system are shown (green line: slope = 0.94, *R*^2^ = 0.98 for each). The percent isotopic selectivity toward ^2^H incorporation into the [4]-position of NADH was determined using ^1^H NMR spectroscopy as described in Section S.1.2.2 in the Supporting Information. Experimental details are provided in Section S.1.2.1 in the Supporting Information.

Having established their ability to reduce NAD^+^, we investigated the isotopic selectivities of the H_2_-driven catalysts under various labeling conditions using either ^1^H_2_ or ^2^H_2_ gas in solutions of ^1^H_2_O:^2^H_2_O at different ratios. The results in Figure 3 show that, for the *Bio* and *Chemo-Bio* systems, the extent of ^2^H incorporation is determined exclusively by the percentage of ^2^H in the solvent (^1^H_2_O:^2^H_2_O), irrespective of the reductant gas (^1^H_2_ or ^2^H_2_). The *Chemo* system, on the other hand, shows a marked preference for the introduction of ^1^H, only producing fully labeled cofactor when it was operated under both 100% ^2^H_2_(g) and 100% ^2^H_2_O. These results again demonstrate the lack of selectivity when Pt is the site of NAD^+^ reduction (the *Chemo* system) and the ability of site-separated enzymatic catalysts (the *Bio* and *Chemo-Bio* systems) to prevent ^1^H^+^ ions generated by the oxidation of ^1^H_2_ from being transferred to the NAD^+^ during reduction.

**Figure 4:**
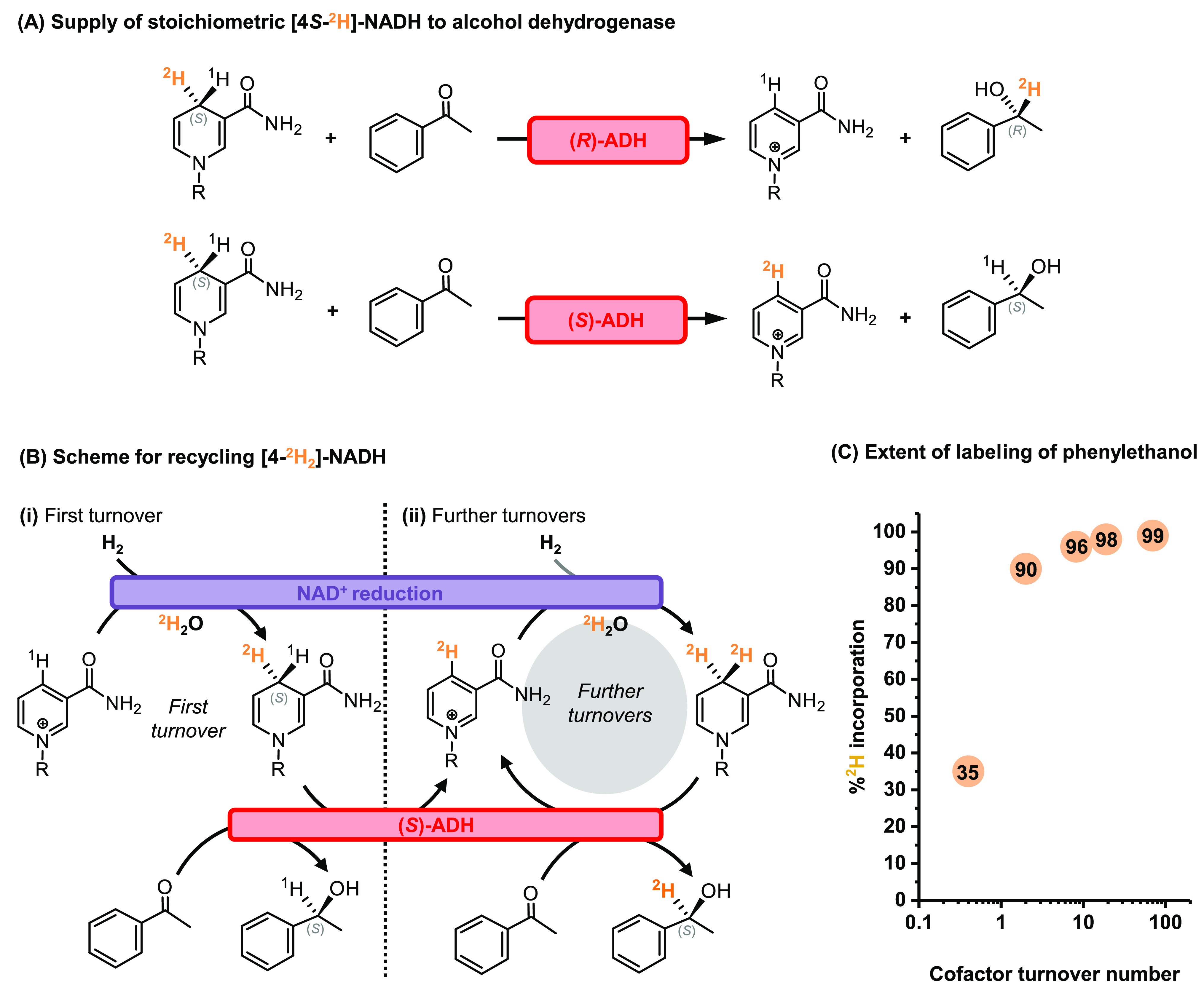
Understanding %^2^H incorporation of enzymes with opposing facioselectivity. (A) When ADH enzymes with opposite facioselectivity are supplied with [4*S*-^2^H]-NADH, ^*x*^H^–^ is removed from either the *S* or *R* face of the cofactor and transferred to the substrate (acetophenone). This leads to generation of either a ^2^H-labeled product or an unlabeled product (with the ^2^H label remaining on the oxidized cofactor). (B) If access to the [*S*-^2^H]-labeled alcohol is required, the cofactor can be recycled *in situ*. The first cofactor turnover leads to unlabeled product (i), and further cofactor turnovers proceed *via* [4-^2^H_2_]-NADH and lead to labeled product (ii). (C) The *Bio* system was used to supply labeled cofactor to (*S*)-ADH with a range of cofactor/substrate ratios, and the resulting solutions were analyzed by ^1^H NMR spectroscopy (see Figure S11 in the Supporting Information). Increasing the cofactor turnover number was found to increase ^2^H incorporation into the phenylethanol product, tending toward 100%. Reactions were conducted on a 0.5 mL scale in ^2^H_5_-Tris-^2^HCl (100 mM, p^2^H 8.0) with 400 μg of the *Bio* system as the catalyst and an excess (500 μg) of (*S*)-ADH being shaken under a steady stream of H_2_ gas at 20 °C. In all of the reactions the initial loading of acetophenone was kept constant at 10 mM, with 2 vol % ^2^H_6_-DMSO as a cosolvent. The starting concentration of NAD^+^ was then varied from 0.1 to 25 mM to give varying turnover numbers, with all reactions giving conversions >70% after 16 h.

The results of the screening revealed that the *Bio*, *Chemo-Bio*, and *Electro-Enzymatic* catalysts are all suitable for selectively making [4*S*-^2^H]-NADH. This compound, which is not currently commercially available, is valuable in its own right, especially for use in enzymatic mechanistic studies and to probe complex biological systems.^1−3,54−63^ However, this form of the cofactor is only suitable for performing reductive deuteration when it is supplied to an NADH-dependent enzyme that removes ^2^H^–^ from the (4*S*)-position of NADH. In order to further explore this phenomenon, two commercial alcohol dehydrogenase (ADH) enzymes were found with opposing facioselectivity toward NADH (Johnson Matthey ADH101 and ADH105; see discussion in Section S.2.4 in the Supporting Information). These ADH enzymes also have opposing selectivities in their reduction of acetophenone to (*R*)- or (*S*)-1-phenylethanol; hence, they are abbreviated as (*R*)-ADH and (*S*)-ADH accordingly. When (*R*)-ADH is supplied with the [4*S*-^2^H]-labeled cofactor, the deuterium label is selectively transferred to the substrate (acetophenone) to produce a product ((*R*)-1-phenylethanol) labeled with ^2^H at the α-position, plus oxidized cofactor (see Figure 4A). However, when [4*S*-^2^H]-NADH is instead supplied to (*S*)-ADH, the unlabeled alcohol product is formed along with the labeled oxidized cofactor [4-^2^H]-NAD^+^ (see Figure 4A). For this reason, access to [4*R*-^2^H]-NADH is needed for enzymes that abstract ^*x*^H^–^ from the (4*R*)-position of the nicotinamide cofactor (such as (*S*)-ADH). Given that no NAD^+^ reductase moiety was available with the desired selectivity for forming [4*R*-^2^H]-NADH, we explored a different strategy for suppling oxidoreductases with such facioselectivity. The alternative approach was based on the effects of undergoing multiple cofactor turnovers, enabling [4-^2^H_2_]-NADH generation, as explained in the following section.

Under turnover conditions, (*R*)-ADH takes ^*x*^H^–^ from the (4*S*)-position of the labeled nicotinamide ring, and the cofactor simply cycles between NAD^+^ and [4*S*-^2^H]-NADH. Thus, each pass through the catalytic cycle produces a labeled alcohol product, and high %^2^H incorporation is expected regardless of turnover number (defined as the number of times each cofactor molecule is used to perform acetophenone reduction). When (*S*)-ADH is employed, ^*x*^H^–^ is taken from the (4*R*)-position of the labeled nicotinamide ring, and the first cofactor turnover therefore produces unlabeled phenylethanol and leaves the deuterated oxidized cofactor [4-^2^H]-NAD^+^ (Figure 4B(i)). The second pass through the cofactor recycling system then yields the doubly deuterated reduced cofactor [4-^2^H_2_]-NADH, and therefore the second turnover of the (*S*)-ADH generates a labeled alcohol product (Figure 4B(ii)). The [4-^2^H]-NAD^+^/[4-^2^H_2_]-NADH cycling then continues by the same means, and subsequent (*S*)-ADH turnovers form [1*S*-^2^H]-phenylethanol. When the cofactor/substrate ratio is lowered, the cofactor turnover number increases, thereby giving a higher proportion of ^2^H-labeled product, as shown by ^1^H NMR analysis (Figure S11 in the Supporting Information). As the cofactor turnover number becomes sufficiently high (>10), the extent of ^2^H incorporation into the product approaches 100% (see Figure 4C). This result demonstrates the versatility of the *Bio* system to drive deuteration reactions by NADH-dependent enzymes regardless of their facioselectivity. Correspondingly, the *Chemo-Bio* system was found to replicate the chemoselectivity, stereoselectivity, and isotopic selectivity of the *Bio* system when it was tested in similar C=O and C=C bond reductive deuteration reactions (see Section S.2.6 in the Supporting Information).

In summary, we have demonstrated multicomponent catalytic systems for the preparation of deuterium-labeled nicotinamide cofactors that rely on ^2^H_2_O as the deuterium source and use either an electrode or H_2_ gas to supply electrons. While the choice of H_2_ oxidation catalyst is flexible (enzyme or metal), the results show that, for the reductive deuteration of NAD^+^ to be achieved with high chemoselectivity and isotopic selectivity, the use of an NAD^+^ reductase enzyme is essential. The NAD^+^ reductase also ensures stereoselectivity toward the formation of exclusively [4*S*-^2^H]-NADH. This [4*S*-^2^H]-NADH can subsequently be used to drive NADH-dependent enzymes to generate [^2^H]-labeled products with high %^2^H irrespective of their facioselectivity, provided that the cofactor turnover number is sufficiently high (≥10).

The principles that underpin the existing *Bio*-catalyst deuteration system have therefore been extended to novel *Electro-Enzymatic* and *Chemo-Bio* catalysts. The newly designed heterogeneous systems overcome the known difficulties of implementing hybrid catalysts, particularly those associated with the combination of metals and enzymes. Crucially, we found that the selectivity of the *Chemo-Bio* catalyst could be tuned by varying the enzyme/metal ratio.

It is interesting to note the similarities of the catalyst systems designed here and reports by other authors of *in vivo* deuteration of hydride transfer cofactors in ^2^H_2_O.^55^ In both cases, facile hydrogen isotope exchange at a flavoprotein active site enables deuterons from the bulk solution to be transferred to the nicotinamide ring, regardless of the identity of the supplied reducing agent (H_2_, electrochemical, or organic).

Together, the *Bio*, *Electro-Enzymatic*, and *Chemo-Bio* catalysts described here represent three distinct but related strategies for the generation and recycling of deuterated cofactors. It is anticipated that these catalysts will improve the ease with which [^2^H]-labeled NADH can be employed in chemical synthesis and mechanistic studies.

## Supporting Information Available

Details of the experimental methods, catalyst preparation, and analytical protocols, relevant electrochemical and spectroscopic results, and additional schemes and discussions to support the findings in the main body of the text (PDF)
