# Supplementary material for: Hybrid Chemo-, Bio-, and Electrocatalysis for Atom-Efficient Deuteration of Cofactors in Heavy Water
Source: ACS Catal. 2021 Feb 11;11(5):2596–604. doi: 10.1021/acscatal.0c03437 (PMC8025731; doi:10.1021/acscatal.0c03437)
Supplement: Supplementary file 1 — cs0c03437_si_001.pdf [file cs0c03437_si_001.pdf]

Supporting Information

Hybrid Chemo-, Bio-, and Electrocatalysis for Atom Efficient Deuteration of Cofactors in Heavy Water

Jack S. Rowbotham,‡ Holly A. Reeve‡* and Kylie A. Vincent‡*

‡ Department of Chemistry, University of Oxford, Inorganic Chemistry Laboratory, South Parks Road, Oxford, OX1 3QR, United Kingdom

*Correspondence to: holly.reeve@chem.ox.ac.uk, kylie.vincent@chem.ox.ac.uk

Table of contents

List of abbreviations for cofactors 2

S.1 Materials and Methods 3

S.1.1 Solvents, reagents, and catalysts 3

S.1.1.1 General reagents 3

S.1.1.2 Enzymes 3

S.1.1.3 Catalyst preparation 3

S.1.2 Procedures for conducting and analyzing ^{2}H-labeling reactions 4

S.1.2.1 Typical reaction conditions 4

S.1.2.2 Procedure for analyzing reaction products by ^{1}H NMR spectroscopy 5

S.1.2.3 Procedure for analyzing reaction products by HPLC 7

S.1.2.4 Procedure for analyzing reaction products by UV-vis spectroscopy 8

S.2 Supplementary Results and Discussion 8

S.2.1 Screening *Electro-Chemo* and *Electro-Enzymatic* catalyst systems 8

S.2.2 Screening *Chemo*, *Chemo-Bio*, and *Bio* catalyst systems 11

S.2.3 Isotopostereoselectivity of the NAD$^+$ reductase enzyme 14

S.2.4 Cofactor facioselectivity of alcohol dehydrogenases 15

S.2.5 Analyzing the role of cofactor turnover number (ToN) 18

S.2.6 Production of ^{2}H-labeled chemicals by the *Chemo-Bio* system 19

S.3 Supplementary References 23

List of abbreviations for cofactors

**NAD^+**	Nicotinamide adenine dinucleotide (oxidized form)
**[4-^{2}H]-NAD^+**	Nicotinamide adenine dinucleotide (oxidized form), deuterated at 4-position of nicotinamide ring
**NADH**	Nicotinamide adenine dinucleotide (reduced form)
**[4-^{2}H]-NADH**	Nicotinamide adenine dinucleotide (reduced form), singly deuterated at 4-position of nicotinamide ring. Unspecified stereochemistry.
**[4*R*-^{2}H]-NADH**	Nicotinamide adenine dinucleotide (reduced form), singly deuterated at 4-position of nicotinamide ring. (*R*)-form.
**[4*S*-^{2}H]-NADH**	Nicotinamide adenine dinucleotide (reduced form), singly deuterated at 4-position of nicotinamide ring. (*S*)-form.
**[4-$^2H_2$]-NADH**	Nicotinamide adenine dinucleotide (reduced form), doubly deuterated at 4-position of nicotinamide ring.

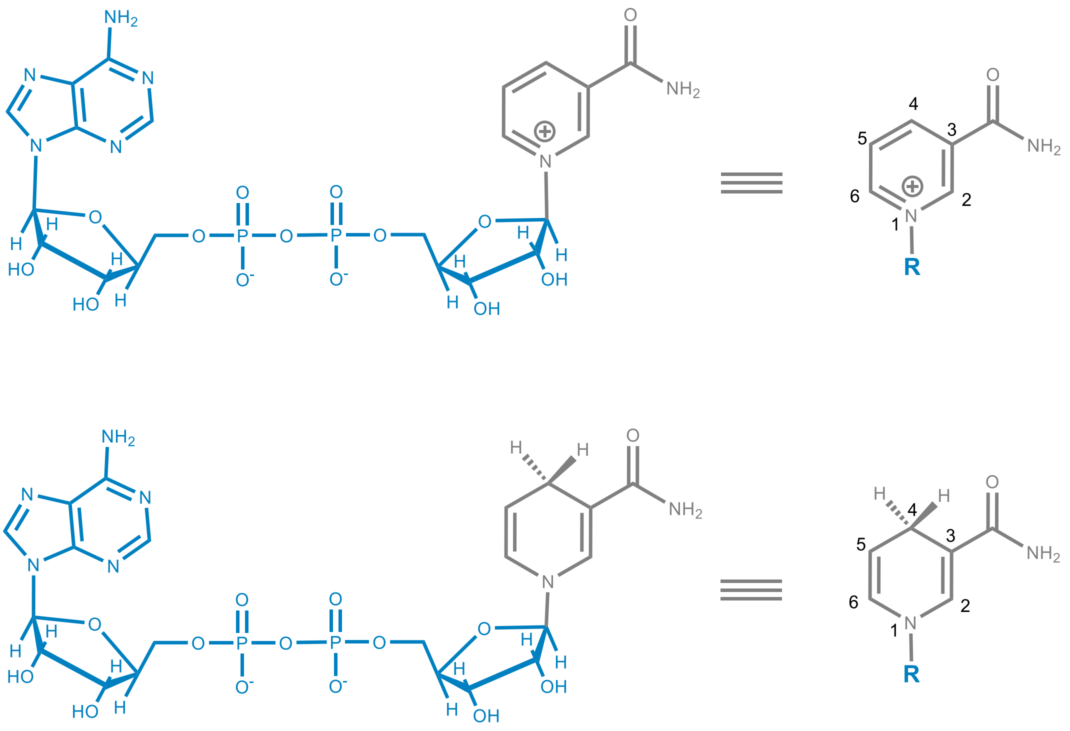

Fig. S 1 NAD^+ (upper) and NADH (lower) in full and abbreviated forms

S.1 Materials and Methods

S.1.1 Solvents, reagents, and catalysts

S.1.1.1 General reagents

All commercial reagents were used as received and without further purification, unless specified otherwise. NAD^+ and NADH were purchased from Prozomix, Pt/C (HiSPEC^{TM} 3000, nominally 20 wt%) from *Alfa Aesar*, carbon black particles (Black Pearls 2000, BP2000) from Cabot Corporation, and all others from Sigma Aldrich. Deionised MilliQ water (Millipore, 18 MΩcm) was used for non-deuterated solutions, and ^2H_2O (99.98 %, Sigma Aldrich) was used for deuterated solutions. Deuterated buffers were prepared as described previously.[1] All aqueous solutions were sparged with dry N_2 for 60 minutes prior to use in order to deoxygenate them.

S.1.1.2 Enzymes

The hydrogenase (*Escherichia coli hydrogenase* 1, molecular weight 100 kDa per HyaAB unit), and NAD^+ reductase (I64A variant of the soluble hydrogenase from *Ralstonia eutropha*, in which the inherent hydrogenase moiety is inactivated, molecular weight 170 kDa)[2] were isolated and purified following published protocols.[3] Commercial samples of alcohol dehydrogenases (*R*)-ADH (ADH101) and (*S*)-ADH (ADH105) and ene-reductase (ENE101) (Johnson Matthey) were received in their lyophilised forms and used without further purification. The (*R*)- and (*S*)-descriptors of the ADH enzymes refer to their selectivities in the reduction of acetophenone to form the corresponding enantiomers of 1-phenylethanol, as determined previously.[4]

S.1.1.3 Catalyst preparation

All catalysts were prepared in a glovebox in deoxygenated (non-deuterated) Tris-HCl buffer (100 mM, pH 8.0) under a protective N_2 atmosphere (O_2 < 0.1 ppm). When catalysts were required for deuteration reactions, they were exchanged into ($^2H_5$)-Tris-^{2}HCl (100 mM, p^{2}H 8.0) by centrifugation (10 000 × g, 5 mins), removal of the supernatant, and re-suspension. For all of the enzymatic catalysts, the loss of the brown/amber colouring of the supernatant from the starting solution was a sign of the high immobilization of the enzyme, further evidenced by the disappearance of solution enzyme activity as determined by standard benzyl viologen assays.[5]

***Electro-Chemo* system** (Pt/C on graphite electrode): prepared by sonication of a 20 mg mL^{-1} suspension of platinum on activated carbon (nominally 20 wt%) in Tris-HCl (100 mM, pH 8.0) for 5 × 15 minutes (with agitation of the solution in between). An aliquot (1 μL) of the Pt/C solution was then dried on to the surface of a freshly polished pyrolytic graphite 'edge' rotating disc electrode. The additional carbon support enabled high catalyst loadings on a small geometrical electrode area.

***Electro-Enzymatic* system** (NAD^+ reductase on carbon on graphite electrode): prepared by sonication of a 20 mg mL^{-1} suspension of BP2000 carbon black in Tris-HCl buffer for 5 × 15 minutes (with agitation of the solution in between). An aliquot of the carbon suspension was then added to an equal volume of NAD^+ reductase (1.4 mg mL^{-1}) and a small amount (1 μL) of the resulting mixture was partially dried on to the surface of a freshly polished pyrolytic graphite edge rotating disc electrode, taking care not to fully dry out the protein.

***Bio* system** (hydrogenase and NAD^+ reductase on carbon): prepared by sonication of a 20 mg mL^{-1} suspension of BP2000 carbon black in Tris-HCl buffer for 5 × 15 minutes (with agitation of the solution in between). Equal volumes of hydrogenase (4 mg mL^{-1}) and NAD^+ reductase (1.4 mg mL^{-1}) were then pre-mixed together, and added immediately to an aliquot of sonicated carbon. After standing at 4 ˚C for 60 minutes, the solid catalyst was then removed from the preparatory suspension by centrifugation (10, 000 × g, 5 mins) before being re-suspended in deuterated or non-deuterated buffer as required. Enzyme loadings: hydrogenase loading of 333 pmoles per 100 μg of carbon, NAD^+ reductase loading of 110 pmoles per 100 μg of carbon.

***Chemo-Bio* system** (platinum and NAD^+ reductase on carbon): prepared by sonication of a 20 mg mL^{-1} suspension of Pt/C (nominally 20 wt%) in Tris-HCl (100 mM, pH 8.0) for 5 × 15 minutes (with agitation of the solution in between). An aliquot of NAD^+ reductase (1.4 mg mL^{-1}) was subsequently added to the necessary volume of Pt/C suspension and allowed to stand at 4 ˚C for 60 minutes. The solid catalyst was then removed from the preparatory suspension by centrifugation (10, 000 × g, 5 mins) before being re-suspended in deuterated or non-deuterated buffer as required. Catalyst loading: NAD^+ reductase 110 pmoles per 100 μg of carbon and Pt 100 nmoles per 100 μg carbon (equating to an enzyme to metal mass ratio of approximately 1.0). Other enzyme/metal mass ratios were explored (see Section S.2.2).

***Chemo* system** (Pt/C): prepared by sonication of a 20 mg mL^{-1} suspension of platinum on carbon (nominally 20 wt%) in Tris-HCl (100 mM, pH 8.0) for 5 × 15 minutes (with agitation of the solution in between). The catalyst suspension was stood for 20 hours prior to centrifugation (10, 000 × g, 5 mins), removal of the liquid, and resuspension in deuterated or non-deuterated buffer as required. Catalyst loading: Pt 100 nmoles per 100 μg carbon.

S.1.2 Procedures for conducting and analyzing ^{2}H-labeling reactions

S.1.2.1 Typical reaction conditions

Electrode-driven reaction: An electrochemical cell was set up to allow for bulk electrolysis reactions. The cell contained a pyrolytic graphite edge rotating disc working electrode[6] (coated in the appropriate catalyst as described above), a saturated calomel reference electrode (SCE), and a platinum wire (0.4 mm diameter) counter electrode, all connected to a potentiostat. The rotation of the working electrode (3000 rpm) was sufficient to ensure the reaction mixtures were well mixed, without the need for additional stirring. In order to prevent reaction of the cofactor molecules at the counter electrode, the platinum wire was separated from the rest of the solution by means of a plastic sheath tipped with a molecular sieve frit. The deoxygenated deuterated buffer was added ([$^2H_5$]-Tris.^{2}HCl, 5 mL, 50 mM, p^{2}H 8.0) and a cyclic voltammogram (10 mV s^{-1}, electrode rotation 3000 rpm) was recorded over the potential range under investigation in order to provide a “blank” trace. The NAD^+ was then added to the solution (4 mM) and mixed thoroughly, prior to the re-recording of the cyclic voltammogram to establish the presence of a catalytic current above any background current observed in the blank. Following initial cyclic voltammetry, the electrode was poised at a chosen potential and the current recorded over 8 hours

(chronoamperometry). A small aliquot of the reaction solution was collected before and after the potential was applied, and analyzed using the methods described in **SI Sections S.1.2.2 - S.1.2.4**.

H_2-driven reactions: All reactions were set up in a glovebox under a protective N_2 atmosphere (O_2 < 0.1 ppm) and were conducted on a 500 μL scale in sealed 0.5 mL micro-centrifuge tubes (Eppendorf) punctured with five holes in the lid (Ø 1.0 mm). Solid catalysts were added at a loading of 100 - 400 μg carbon mL^{-1}. Reaction mixtures were pre-saturated with reductant gas ($^1H_2$ or $^2H_2$) and the cofactor dissolved prior to the addition of the catalyst, to ensure that both enzymes were exposed to substrate simultaneously. In order to help dissolve organic compounds ($^2H_6$)-dimethylsulfoxide (DMSO) was included in the reactions as required (1-5 vol.%). Deuterated DMSO was used instead of unlabelled DMSO for analytical purposes, and these ^{2}H atoms did not play a part in the reactions.

The sealed tubes were then transferred to specially adapted shaker plate, which allowed for a steady flow of H_2 across the headspace. Alternatively, if a pressurized H_2-atmosphere was required, a Tinyclave pressure vessel (BUCHI) was used, and rocked back and forth at 15 rpm whilst the reactions took place. Following the reaction, solid catalyst was removed by two rounds of centrifugation (10, 000 × g, 5 mins), and the solution was analyzed by the methods described in **SI Sections S.1.2.2 - S.1.2.4**.

S.1.2.2 Procedure for analyzing reaction products by ^{1}H NMR spectroscopy

Following removal of the catalyst particles by filtration or centrifugation, 450 μL of the sample solution was transferred to a Norell® SelectSeries™ 5 mm 400 MHz sample tube. A further 50 μL of ^2H_2O was added for field locking purposes and, when required, 0.5 mM of acetone was also included to act as a reference.

^{1}H NMR spectroscopy was carried out on either a Bruker Avance III HD nanobay (400 MHz) or Bruker Avance III (500 MHz), with samples for direct comparison always being run on the same machine. Both machines were equipped with 5 mm z-gradient broadband multinuclear probes. Spectra were acquired at 293 K using standard 1-dimensional techniques, including water suppression (Bruker *noesygppr1d* pulse sequence) as required (full Spectrometer parameters are tabulated elsewhere).[1] Data were processed in MestReNova after application of the Bruker *proc_1d* or *proc_1dakps* processing algorithms, with manual re-phasing being conducted where necessary. A multipoint baseline correction was also applied across the entirety of the spectral window and a line broadening corresponding to 0.5 Hz was applied to each spectrum to improve the S/N ratio. Signals were referenced against an appropriate signal: acetone (δ = 2.22 ppm), ($^2H_5$)-dimethylsulfoxide (δ = 2.71), or the Tris peak (δ = 3.70 ppm), all of which were referenced originally to 4,4-dimethyl-4-silapentane-1-sulfonic acid (δ = 0.00 ppm).

The spectra of nicotinamide cofactors and their deuterated analogues were assigned according to well-established literature arguments.[7,8] The peak(s) between 2.64 – 2.84 ppm (corresponding to the 4-position of the dihydronicotinamide ring, **Fig. S 1**) were used to discern relative abundance of the various isotopologues of NADH according to the method in **Fig. S 2** and accompanying equations.

In order to verify that the relationships in **Fig. S 2** were independent of the isotopic constitution of the sample solvent, an additional calibration was carried out. Here, a 3 mM sample of NADH was prepared in solutions of varying %^2H_2O (from 100 vol.% ^1H_2O to 100 vol.% ^2H_2O). The relative areas of peaks **A**, **B**, **C**, and **D** measured in each solution were plotted (**Fig. S 3**) and, within experimental error, the isotopic constitution of the NMR solvent was found to have no bearing on the result.

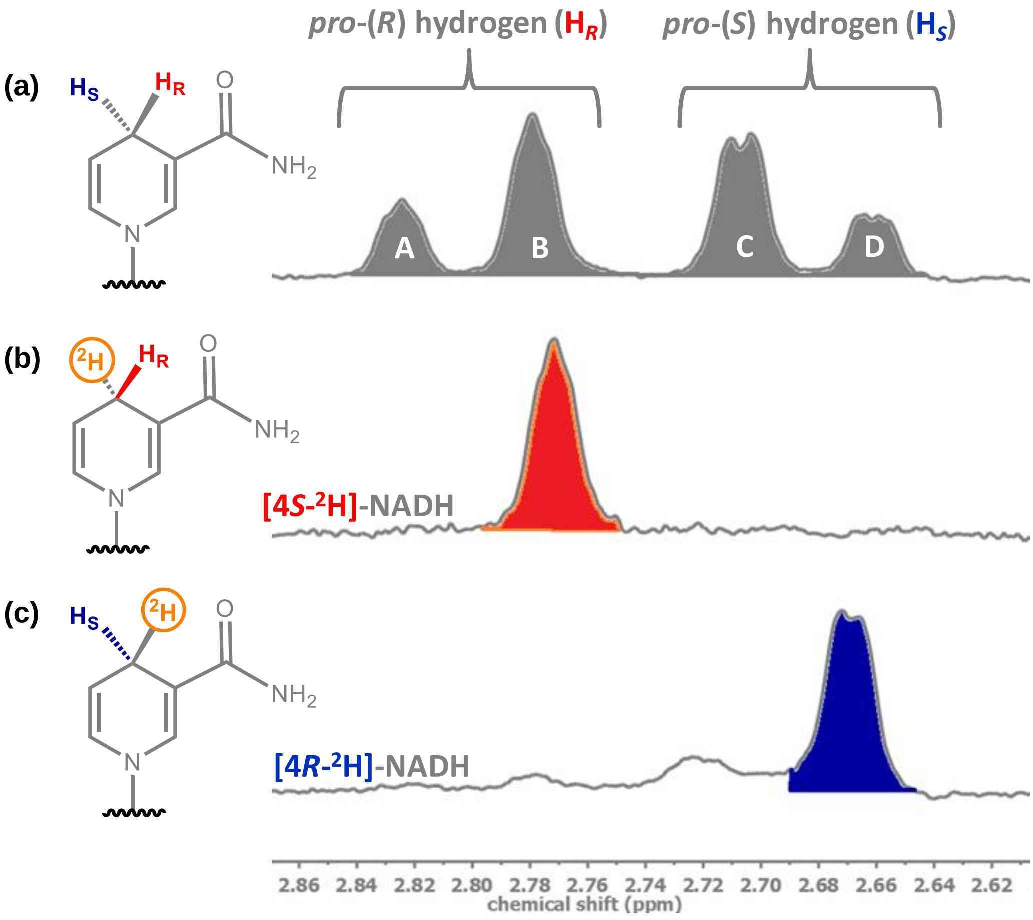

(d)

$$\%\ [^2H]\text{-NADH} = \frac{B - C + D - A}{B + D} \times 100$$

$$\%\ [4S\text{-}^2H]\text{-NADH} = \frac{B - C}{B + D} \times 100$$

$$\%\ [4R\text{-}^2H]\text{-NADH} = \frac{D - A}{B + D} \times 100$$

Fig. S 2 Diagnostic signal for the interpretation of ^1H NMR spectra of reduced nicotinamide cofactors containing different deuterium labels (^2H_2O, p^{2}H 8.0, 400 MHz, 293 K). **(a)** NADH at natural isotopic abundance, **(b)** [4*S*-^{2}H]-NADH, and **(c)** [4*R*-^{2}H]-NADH. **(d)** The equations used to quantitatively determine the proportion of each form of the cofactor, where **A**, **B**, **C**, and **D** represent the integrals of the peaks defined in **(a)**.

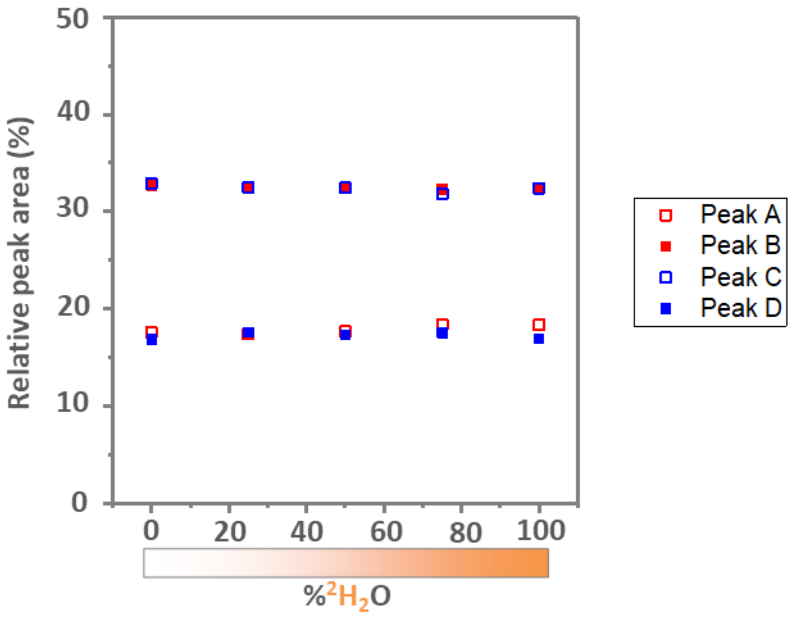

Fig. S 3 Results from a control experiment to determine the variation of peak areas in the ^{1}H NMR spectra of unlabeled NADH acquired in aqueous solutions of varying %^2H_2O (p^{2}H 8.0, 400 MHz, 293 K). Within error, it can be seen that the areas of diagnostic peaks **A**, **B**, **C**, and **D** (defined in **Fig. S 2**) are reproducible across all of the solvent mixtures, and are unaffected by the differing levels of solvent signal suppression used.

S.1.2.3 Procedure for analyzing reaction products by HPLC

All HPLC was conducted on a Shimadzu UFLC LC-20AD Prominence liquid chromatograph equipped with a dual wavelength UV-spectrophotometric detection. MilliQ water and HPLC grade solvents were used throughout. The HPLC method was as follows, unless specified otherwise:

Description: Hydrophilic Interaction Liquid Chromatography (HILIC) for cofactor analysis
Pre-treatment: Reactions containing just NAD^+/NADH were first filtered through a nylon syringe filter (Gilson, 0.22 μm), and then centrifuged at 18, 800 × g for 5 minutes to remove any smaller particulates. Samples were transferred to glass HPLC vials without further dilution.
Column: SeQuant® ZIC®-HILIC, 5 μM particle size, 200 Å pore size, 20 × 2.1 mm bed
Buffer A: 90 vol.% acetonitrile (Honeywell, CHROMASOLV® 99.9%): 10 vol.% MilliQ water, 20 mM ammonium acetate, pH 7.5
Buffer B: 100 vol.% MilliQ water, 20 mM ammonium acetate, pH 7.5
Column temperature = 40 ˚C
Flow rate = 1 mL min^{-1}
Injection volume = 10 μL
Detection = 260 and 340 nm
Solvent profile: 0 → 1 mins: 100% A, 0% B (isocratic); 1 → 31 mins: 100% A, 0% B to 20% A, 80% B (linear gradient), 31 → 33 mins: 20% A, 80% B (isocractic), 33 → 35 mins: 100% A, 0% B (isocratic).

S.1.2.4 Procedure for analyzing reaction products by UV-vis spectroscopy

Reactions which resulted in changes in the relative concentration of NAD^+/NADH could be followed be UV-vis spectroscopy. Typically, solid particulates were removed by filtration or centrifugation (as described above) and the sample diluted in MilliQ (^1H_2O) water so that the cofactors were in the range 0.1 – 0.2 mM. A background spectrum of pure MilliQ was subtracted from that acquired for the sample. Measurements were made in a quartz cuvette (path length 1 cm, Hellma) on a Cary 60 UV/Vis spectrophotometer (Agilent). The ratio of NAD^+ to NADH could then be determined by measuring the ratio $A_{260\ nm} : A_{340\ nm}$, as described previously.[2]

S.2 Supplementary Results and Discussion

S.2.1 Screening *Electro-Chemo* and *Electro-Enzymatic* catalyst systems

Electrochemical reduction and deuteration of NAD^+ by each of the *Electro-Enzymatic* and *Electro-Chemo* catalysts was investigated according to the procedure described in **Section S.1.2.1**. The electrochemical traces from these investigations are shown in **Fig. S 4**, and the analysis of the products by UV/Vis and HPLC are shown in **Fig. S 5**.

The *Electro-Enzymatic* system shows considerable catalytic reduction current in the presence of NAD^+ (**Fig. S 4 (a)** and **(b)**), with an onset potential of around -340 mV *versus* SHE (no catalytic current was detected in the absence of NAD^+). Chronoamperometry at -560 mV *versus* SHE (**Fig. S 4 (d)**) was used to drive NAD^+ reduction over an 8 hour reaction, and the *Electro-Enzymatic* system gave rise to NADH, evidenced by UV/Vis (**Fig. S 5 (a)** and **(b)**) and HPLC (**Fig. S 5 (d)**). Further analysis by ^1H NMR (see **SI Section S.1.2.2**) showed that only [4*S*-^{2}H]-NADH was formed.

The *Electro-Chemo* system (Pt/C on an electrode) shows a catalytic reduction current at -560 mV, with a more negative onset potential than in the *Electro-Enzymatic* case. The catalytic current is present both with and without NAD^+, and chronoamperometry at this potential (**Fig. S 4 (c)**) does not give rise to detectable product formation by UV/Vis (**Fig. S 5 (a)** and **(b)**). Hence, the large catalytic currents observed for the Pt/C system are attributed to deuteron ($^2H^+$) reduction. Poising the electrode at lower potentials (-760 mV *versus* SHE) does give rise to a small amount of NAD^+ reduction, but analysis by UV/Vis (**Fig. S 5 (a)** and **(b)**) and HPLC (**Fig. S 5 (c)**) show that very little product is 1,4-NADH, and the major additional peak can be attributed to one of six possible NAD_2 dimers.[9–11]

The unmodified (bare) carbon electrode does not show catalytic current in the presence of NAD^+ across the potential window investigated (**Fig. S 4 (a)**). Application of a more negative potential (**Fig. S 4 (c)**) gave rise to a yellowing of the solution, indicative of the degradation of the cofactor to non-biological dimeric forms.

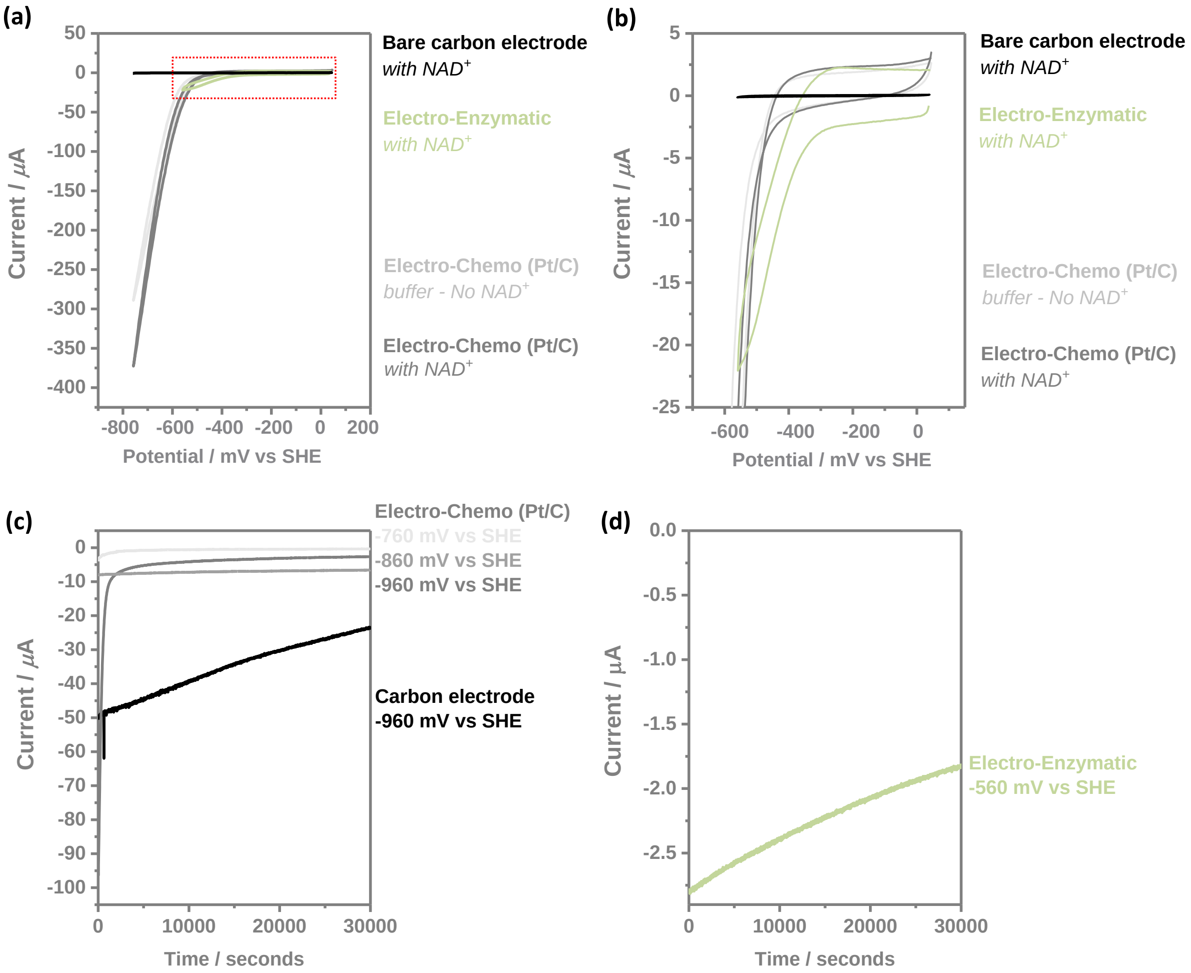

Fig. S 4 Traces from the electrochemical experiments in the presence of NAD+ in ^2H_2O for a range of electrode systems. **(a)** Cyclic voltammograms (10 mV s^{-1}) for the bare carbon electrode and *Electro-Enzymatic* and *Electro-Chemo* catalytic systems in the presence (or absence) of NAD+. The highlighted region in **(a)** is expanded in **(b)**. The traces show a catalytic current for the *Electro-Enzymatic* and *Electro-Chemo* (Pt/C) systems at -560 mV (*versus* SHE) in the presence of NAD+. A similar current for the *Electro-Chemo* system is also observed in the absence of NAD+, indicating that significant deuteron reduction is occurring. The bare carbon electrode does not show any catalytic current down to -960 mV (*versus* SHE). **(c)** Chronoamperometry traces for the *Electro-Chemo* system at different potentials and bare carbon electrode at -960 mV (*versus* SHE), and **(d)** chronoamperometry trace for the *Electro-Enzymatic* system at -560 mV (*versus* SHE). The reactions were carried out in deoxygenated deuterated buffer ([$^2H_5$]-Tris.^{2}HCl, 5 mL, 50 mM, p^{2}H 8.0) in a bulk electrolysis cell with stirring at 20 °C, with NAD+ (4 mM) included in the reaction where specified.

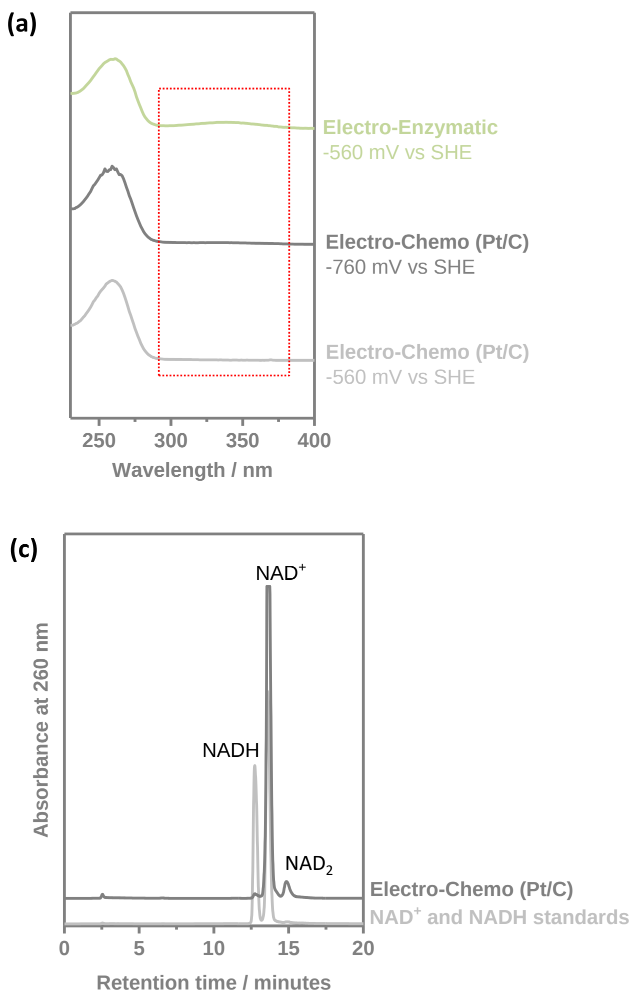

Fig. S 5 Analysis of the products of electrocatalytic reduction of NAD^+. **(a)** UV/Vis analysis of the products of the *Electro-Enzymatic* and *Electro-Chemo* reactions. The highlighted region in **(a)** is expanded in **(b)**. The *Electro-Enzymatic* reaction at -560 mV (*versus* SHE) gives rise to a peak at 340 nm, indicating NADH formation, whilst the *Electro-Chemo* reaction shows no products until a lower potential (-760 mV *versus* SHE) is applied. **(c) and (d)** HPLC analysis of the products of the *Electro-Chemo* reaction at -760 mV *versus* SHE **(c)**, and the *Electro-Enzymatic* reaction at -560 mV *versus* SHE **(d)**. In the *Electro-Chemo* case, there is considerable side-product formation, which is likely a biologically inactive NAD_2 dimer.

S.2.2 Screening *Chemo*, *Chemo-Bio*, and *Bio* catalyst systems

The *Chemo*-, *Chemo-Bio*-, and *Bio*- catalysts were all screened initially for their ability to generate the correct form of NADH (1,4-NADH) when supplied with $^{1}H_2$ gas. Here, 100 μg of the relevant catalyst was shaken with 4 mM NAD^+ ([$^{2}H_5$]-Tris.^{2}HCl, 100 mM, p^{2}H 8.0) for 16 hours under 2 bar $^{1}H_2$. The *Chemo* and *Chemo-Bio* systems proceeded to >90% conversion, and the *Bio* system to around 60% (though additional experiments demonstrated that the latter system could proceed to >90% conversion without change in selectivity). Subsequent HPLC-UV (λ = 260 nm) analysis (**Fig. S 6 (a)(i)**) illustrates that the *Bio* system form only 1,4-NADH, where the *Chemo* system also gives rise to significant quantities of a side product (retention time 9.6 minutes). The side product was confirmed as 1,6-NADH by reducing NAD^+ with $NaBH_4$ to generate a mixture of NADH isomers,[12] which was analysed by HPLC and assigned using the known UV absorptions.[10] The chemoselectivity for 1,4-NADH:1,6-NADH could then be quantified by using the peak areas of the HPLC traces (λ = 260 nm), after correcting for the difference in molar extinction coefficients at that wavelength.[10] Additionally, ^{1}H NMR spectroscopic analysis of the same experiments (**Fig. S 6 (a)(ii)**) illustrate that the *Bio* and *Chemo-Bio* catalysts form only [4*S*-^{2}H]-NADH, whilst the *Chemo* system forms a mixture of [4*S*-^{2}H]-, [4*R*-^{2}H]-, and unlabeled NADH (following the analytical arguments in **SI Section S.1.2.2**).

In the development of the *Chemo-Bio* catalyst, an experiment to determine the effect of the ratio of NAD^+ reductase : Pt was carried out. Here, a series of catalysts ranging from 0.0 to 2.0 μg of NAD^+ reductase per 1.0 μg of Pt (representing the transition from the full *Chemo* system to the final *Chemo-Bio* system) were tested under the same conditions as previously. The results were analyzed by HPLC and ^{1}H NMR spectroscopy (**Fig. S 6 (b)**).The resulting data demonstrate the increasing chemo- and isotopic selectivity of the system as the proportion of NAD^+ reductase is increased, until a point (around 0.5 μg of NAD^+ reductase per 1.0 μg of Pt) where [4*S*-^{2}H]-NADH was the sole product (corresponding to **Figure 2B** in the main manuscript).

Finally, additional experiments were carried out to ascertain the initial activity of the *Chemo*, *Chemo-Bio*, and *Bio* systems for the reduction of NAD^+ in $^{2}H_2O$ and under $^{1}H_{2(g)}$. The NAD^+ reductase/Pt mass ratio for the *Chemo-Bio* catalyst in this case was 0.5, corresponding to the lowest ratio at which only one product ([4*S*-^{2}H]-NADH*)* is formed. The experiments were conducted in buffered solutions (Tris, 50 mM) of $^{2}H_2O$ at pD 6.0 and pD 8.0, and in pure unbuffered $^{2}H_2O$. The buffers were sparged with N_2 (60 mins) prior to the addition of 0.1 mM of NAD^+. The reaction solution was then added to a quartz cuvette (600 μL), sealed, and then sparged for another 15 mins under a steady flow of $H_{2(g)}$. Upon addition of the catalyst (50 μg), the H_2 delivery needle was moved out of the solution and into the headspace of the cuvette, and the UV spectra was recorded every 30 seconds (see **Fig S 7**). Here it was found that the *Chemo-Bio* catalyst more closely resembles the purely *Bio* system, both in terms of activity and sensitivity to pD. Given that the Pt wt.% loading was identical in the *Chemo* and *Chemo-Bio* experiments, the addition of the NAD^+ reductase can be seen to enhance the activity of the Pt/C alone.

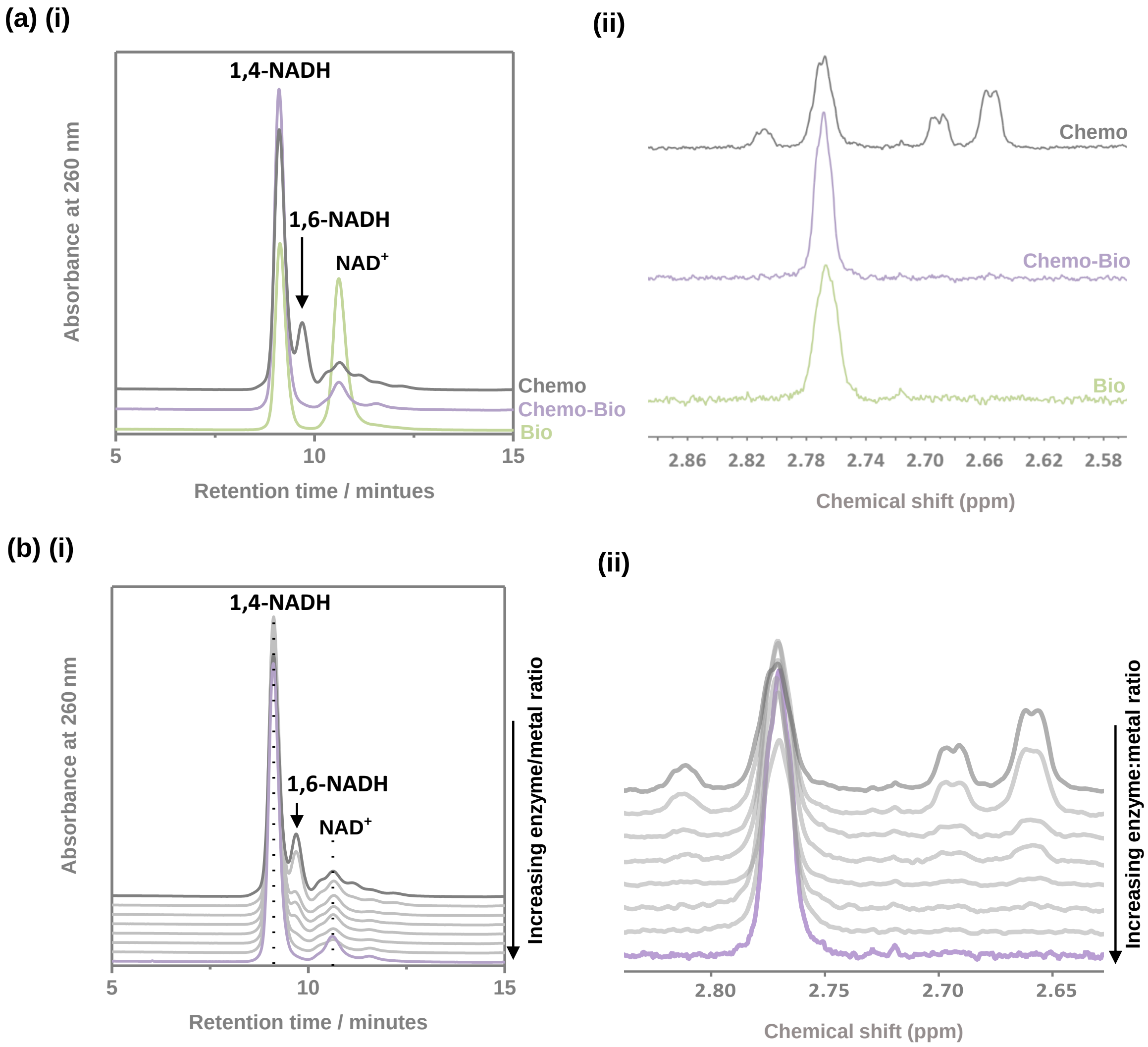

Fig. S 6 (a)(i) HPLC-UV and **(ii)** ^1H NMR spectroscopic analysis (^2H_2O, p^{2}H 8.0, 500 MHz, 293 K) of nictotinamide cofactors following the reduction of NAD^+ by H_2 in the presence of different catalyst systems (*Chemo*, *Bio*, and *Chemo-Bio*). The *Bio* and *Chemo-Bio* systems selectively form [4*S*-^{2}H]-NADH, where the *Chemo* system does not. **(b)(i)** HPLC-UV and **(ii)** ^1H NMR spectroscopic analysis (^2H_2O, p^{2}H 8.0, 500 MHz, 293 K) demonstrating the effects of varying the mass ratio of NAD^+ reductase : Pt on the selectivity of H_2-driven NAD^+ reducation in ^2H_2O. The results show that, as the ratio of NAD^+ reductase increases, the formation of products other than [4*S*-^{2}H]-NADH decreases. Experimental detail provided in **SI Section S.1.2.1**, and ^1H NMR spectroscopic interpretation provided in **SI Section S.1.2.2**.

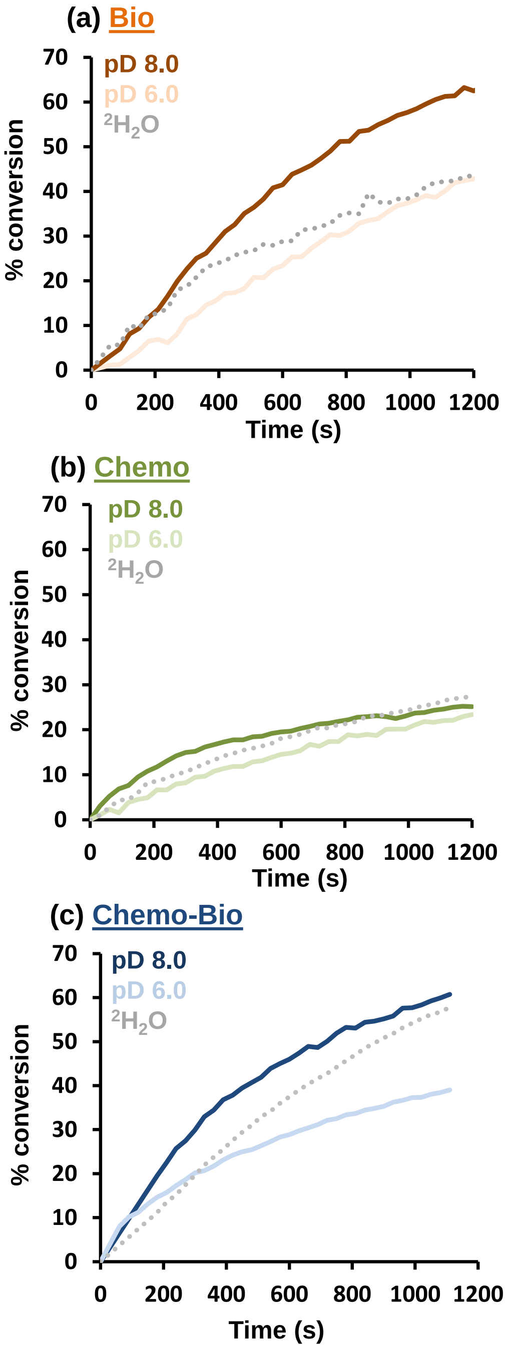

Fig. S 7 Kinetic traces of the reduction of NAD^+ the **(a)** *Bio*, **(b)** *Chemo*, and **(c)** *Chemo-Bio* catalyst systems in ^2H_2O under $^1H_{2(g)}$. The ^2H_2O reaction solution was either unbuffered (gray), or buffered to pD 6.0 (light trace) or pD 8.0 (dark trace). The reactions were carried out in cuvettes with 600 μL of reaction solution (0.1 mM NAD^+) pre-saturated with $^1H_{2(g)}$, and with a headspace of $^1H_{2(g)}$ maintained throughout. A catalyst loading of 50 μg of carbon was used, and an identical amount of Pt was present in the *Chemo* and *Chemo-Bio* systems. The enhancement in activity of the *Chemo-Bio* system is therefore attributable to the addition of the NAD^+ reductase to the Pt/C (at a mass ratio of 0.5 enzyme/metal in this case).

S.2.3 Isotopostereoselectivity of the NAD+ reductase enzyme

The positioning of nicotinamide cofactor relative to the flavin mononucleotide (FMN) active site of the NAD+ reductase moiety, HoxF, was examined *in silico*. No crystal structure is available for the soluble hydrogenase of *Ralstonia eutropha*, HoxHYFU. We therefore prepared a homology model for HoxF using two published crystal structures of the hydrophilic portion of Complex I from *Thermus thermophilus* (PDB accession numbers 2FUG and 3IAM as templates.[13,14] The Nqo1 subunit of *T. thermophilus* Complex I shows 62% sequence similarity with the C-terminal part of HoxF. **Fig. S 8 (a)** and **(b)** confirm that the hydride from FMN is delivered to the *si* face of the cofactor. The homology modelling was performed using SWISS-MODEL.[15] A mechanism is therefore proposed in **Fig. S 8 (c)** to illustrate how the NAD+ reductase utilises electrons and $^2H^+$ from solution to generate a deuteride equivalent ($^2H^-$), which is added to the *si* face of NAD+ to yield the [4*S*-^{2}H]-NADH product.

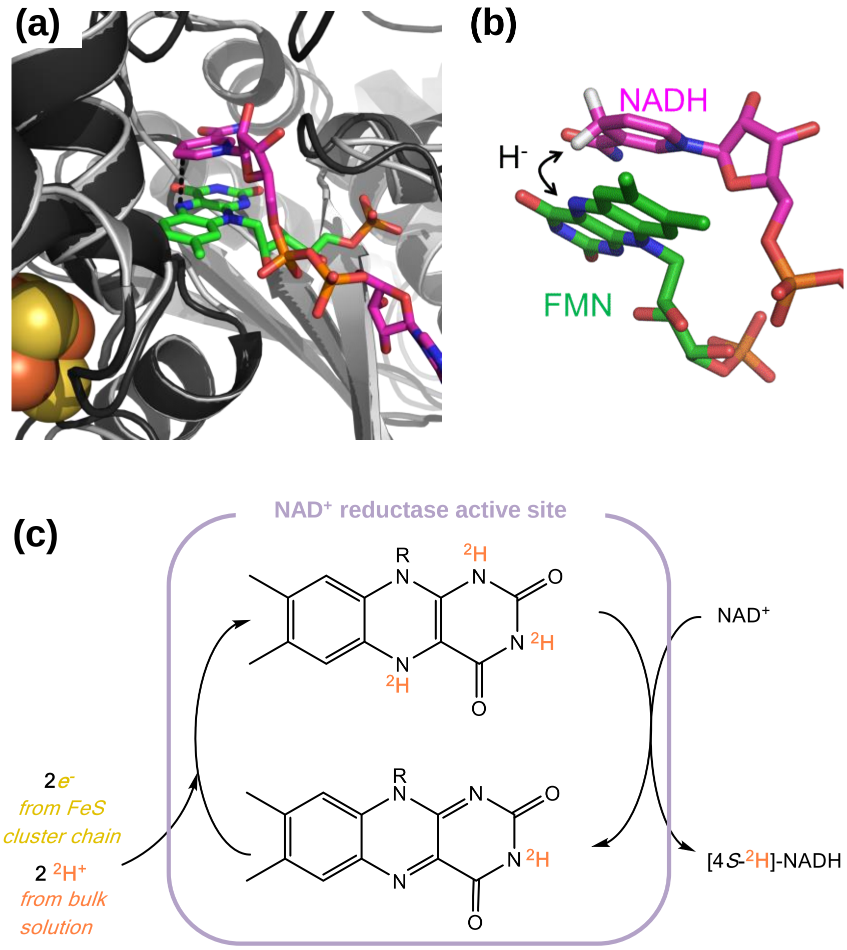

Fig. S 8 (a) X-ray crystal structure of the NAD+ binding site of complex 1 from *Thermus thermophilus* (PDB code: 2FUG; dark gray), overlaid by a homology model for the *R. eutropha* HoxF subunit (light gray). The FMN and NADH structures (shown as sticks) are taken from PDB entry 3IAM - the FMN is underneath the NADH. The black dotted line indicates the atoms in FMN and NADH which are directly involved in hydride transfer, showing that the hydride on FMN will be delivered to the *si*-face of the nicotinamide ring. An expanded view of the NADH and FMN is provided in panel **(b).** The figure was generated using the PyMOL Molecular Graphics System, Version 2.0 Schrödinger, LLC. **(c)** An overview of the mechanism by which the deuteride equivalent ($^2H^-$) is formed and transferred from the FMN cofactor of the NAD+ reductase to an NAD+ molecule to selectively form [4*S*-^{2}H]-NADH.

S.2.4 Cofactor facioselectivity of alcohol dehydrogenases

As explained in the text, different NADH-dependent enzymes have varying selectivities for the face of the dihydronicotinamide ring from which they abstract a hydride (facioselectivity). For NADH of natural isotopic abundance, this selectivity is not manifested in the final product, however, for [4-^{2}H]-NADH the choice determines whether the ^{2}H label is transferred or not. In order to determine the facioselectivites of the two ADH enzymes selected for study ((*R*)- and (*S*)-ADH), a series of stoichiometric reactions were carried out (**Fig. S 9)**. Reactions were performed in 0.5 mL of $^{2}H_2O$ containing [$^{2}H_{11}$]-Tris^{2}HCl (100 mM, p^{2}H 8.0), 10 mM acetophenone (AcPH), 2 vol.% [$^{2}H_6$]-DMSO and 4 mM of the required cofactor (either [4*S*-^{2}H]-NADH prepared using the *Bio* catalyst system, or commercial NADH of natural isotopic abundance). To this mixture, 100 μg of the chosen ADH was added and the progress of the formation of 1-phenylethanol (1-PhEtOH) followed by ^{1}H NMR spectroscopy. Time courses are shown in **Fig. S 10** for the following reactions:

A. (*R*)-ADH + unlabeled NADH + AcPh → (*R*)-1-PhEtOH + NAD^{+} (**Fig. S 10(a)**)
B. (*R*)-ADH + [4*S*-^{2}H]-NADH + AcPh → [1*R*-^{2}H]-PhEtOH + NAD^{+} (**Fig. S 10(b)**)
C. (*S*)-ADH + unlabeled NADH + AcPh → (*S*)-1-PhEtOH and NAD^{+} (**Fig. S 10(c)**)
D. (*S*)-ADH + [4*S*-^{2}H]-NADH + AcPh → (*S*)-1-PhEtOH + [4-^{2}H]-NAD^{+} (**Fig. S 10(d)**)

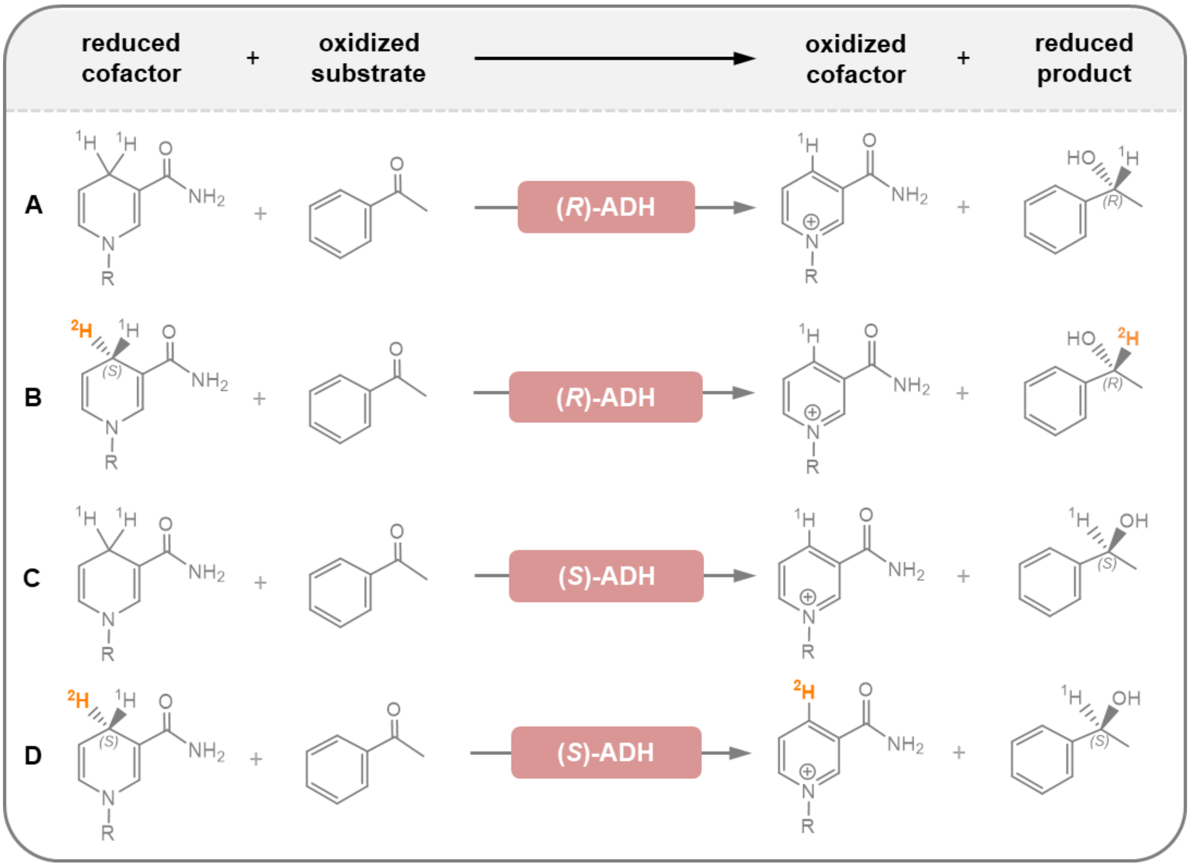

Fig. S 9 Summary of the selectivities of the two commercial ADH enzymes used in this study when provided with substrate (AcPh) and either unlabeled NADH or deuterium labeled [4*S*-^{2}H]-NADH. ^{1}H NMR spectroscopic time courses shown in **Fig. S 10**.

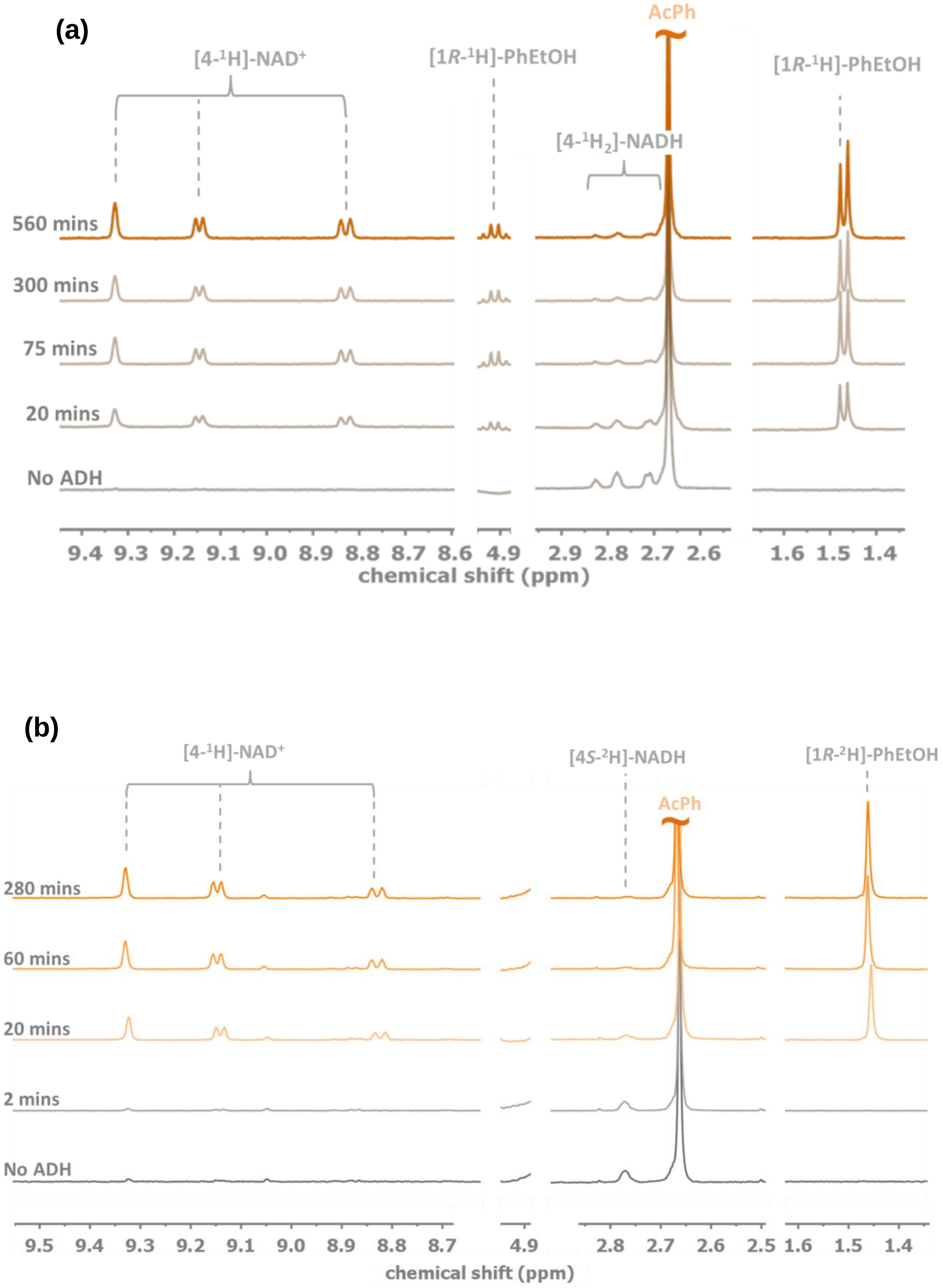
(a)
[4-¹H]-NAD⁺
[1R-¹H]-PhEtOH
AcPh
[4-¹H₂]-NADH
[1R-¹H]-PhEtOH
560 mins
300 mins
75 mins
20 mins
No ADH
9.4 9.3 9.2 9.1 9.0 8.9 8.8 8.7 8.6 4.9 2.9 2.8 2.7 2.6 1.6 1.5 1.4
chemical shift (ppm)
(b)
[4-¹H]-NAD⁺
[4S-²H]-NADH
AcPh
[1R-²H]-PhEtOH
280 mins
60 mins
20 mins
2 mins
No ADH
9.5 9.4 9.3 9.2 9.1 9.0 8.9 8.8 8.7 4.9 2.8 2.7 2.6 2.5 1.6 1.5 1.4
chemical shift (ppm)

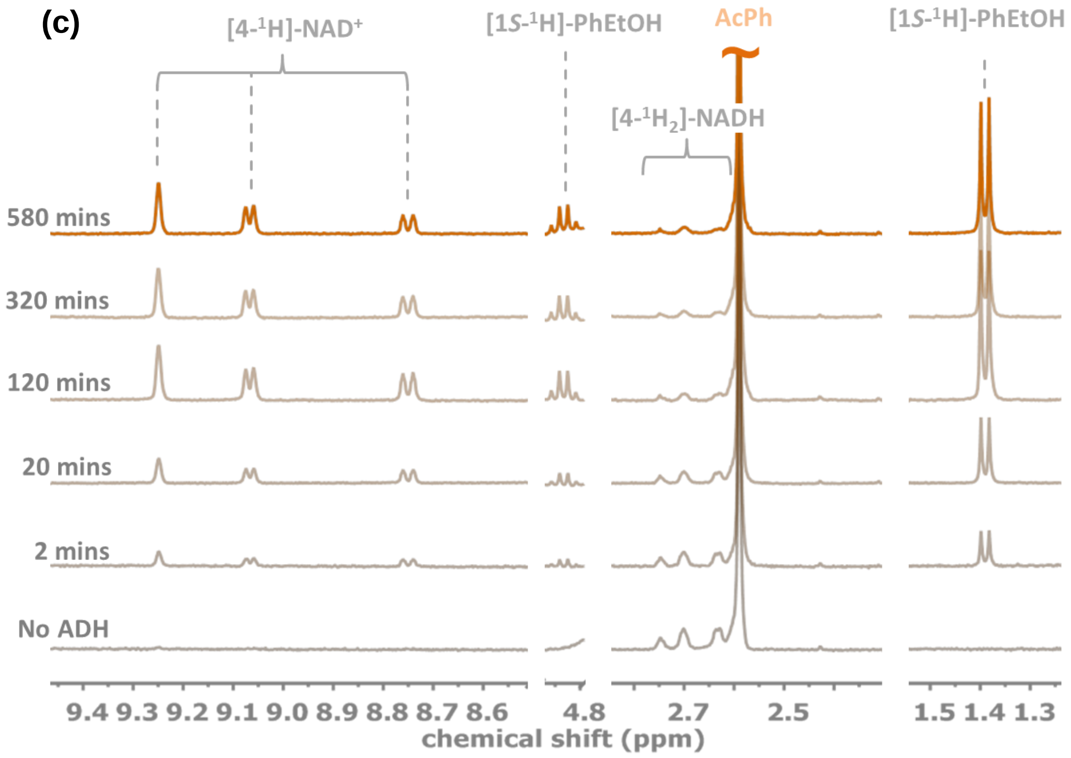

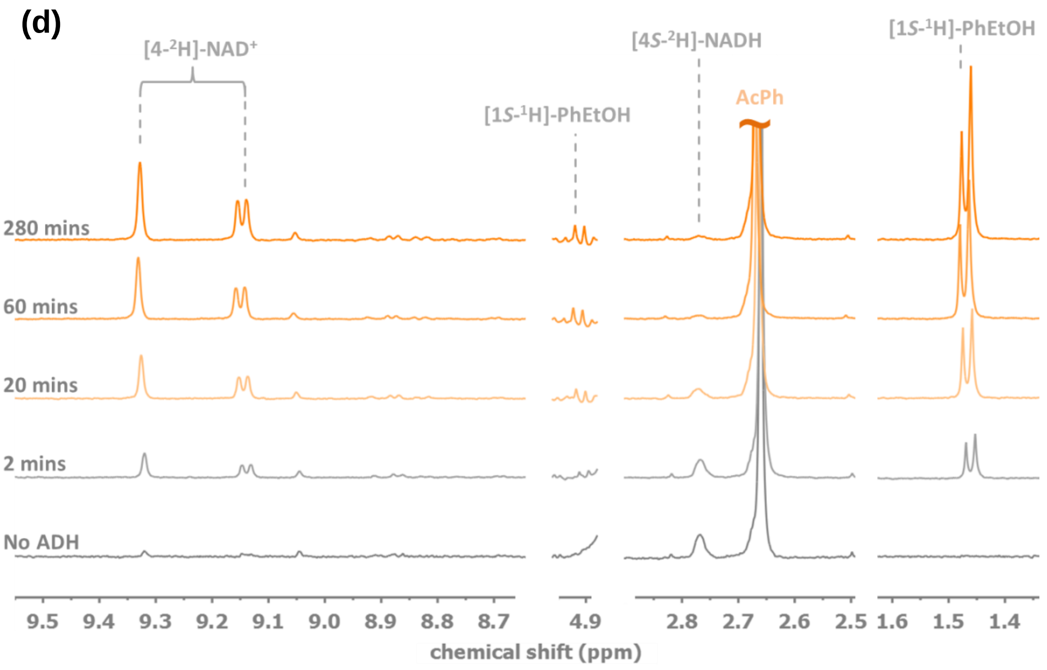

Fig. S 10 ^{1}H NMR spectra ($^{2}H_2O$, p^{2}H 8.0, 400 MHz, 293 K) of reaction mixtures containing excess AcPh with **(a)** unlabeled [4-$^{1}H_2$]-NADH and (*R*)-ADH, **(b)** [4*S*-^{2}H]-NADH and (*R*)-ADH, **(c)** unlabeled [4-$^{1}H_2$]-NADH and (*S*)-ADH, and **(d)** [4*S*-^{2}H]-NADH and (*S*)-ADH.

S.2.5 Analyzing the role of cofactor turnover number (ToN)

As was established in **SI Section S.2.4**, the (*S*)-ADH has the incorrect facioselectivity with regards to NADH in order to transfer the deuterium atom from [4*S*-^{2}H]-NADH to the target compound (acetophenone in this case). However, as was explained in the main manuscript in Figure 4 and associated discussion, multiple turnovers of the cofactor molecule can give rise to the desired [^{2}H]-labelled product. Hence, by maximising the cofactor turnover number, the unlabeled [^{1}H]-labelled product can be "diluted" by subsequent [^{2}H]-product formation. To demonstrate this principle a reaction was carried out with H_2-driven reduction of AcPh by (*S*)-ADH in $^{2}H_2O$ with the cofactor ToN varying from 0.4 to 70. Reactions were performed in 0.5 mL of $^{2}H_2O$ containing [$^{2}H_5$]-Tris.^{2}HCl (100 mM, p^{2}H 8.0) to which 10 mM acetophenone (AcPH), 2 vol.% [$^{2}H_6$]-DMSO and 0.1 - 25 mM of the NAD^+ was added. To this mixture 500 μg of the (*S*)-ADH was added, followed by 400 μg of the *Bio* catalyst. The reaction was shaken at 500 rpm for 16 hours under a flow of $^{1}H_{2(g)}$, prior to removal of the catalysts and analysis by ^{1}H NMR spectroscopy (**Fig. S 11**). All reactions proceeded to ≥ 70% conversion, and the precise cofactor ToN was subsequently determined. Analysis of the peak corresponding to the methyl group of 1-PhEtOH (δ = 1.47 ppm) allowed for a calculation of the %^{2}H at the α-position, according to previous arguments.[1] The results are plotted in the main manuscript, **Figure 4C**.

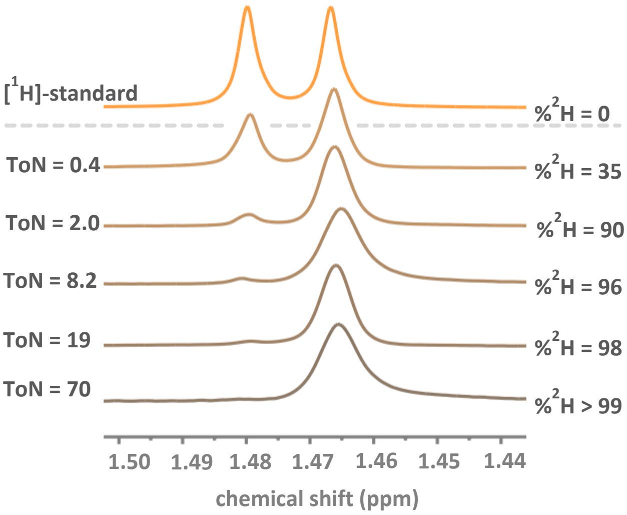

Fig. S 11 ^{1}H NMR spectroscopic analysis ($^{2}H_2O$, p^{2}H 8.0, 500 MHz, 293 K) of the 1-PhEtOH product of the H_2-driven reductive deuteration of AcPh by (*S*)-ADH with different cofactor turnover numbers. The upper trace shows an isotopically pure standard of commercial [1*S*-^{1}H]-PhEtOH, and the lower traces indicate an increasing %^{2}H in the 1-PhEtOH product with increasing cofactor ToN.

S.2.6 Production of ^{2}H-labeled chemicals by the *Chemo-Bio* system

Following successful demonstration of the *Chemo-Bio* system for generating [4*S*-^{2}H]-NADH, it was coupled to a series of NADH-dependent dehydrogenase enzymes in order to demonstrate cofactor recycling to produce [^{2}H]-labelled chemicals as follows:

- With (*R*)-ADH to convert AcPh to [1*R*-^{2}H]-PhEtOH (**Scheme S1**)
- With (*S*)-ADH to convert AcPh to [1*S*-^{2}H]-PhEtOH (**Scheme S2**)
- With ene-reductase to convert cinnamaldehyde to [2,3-$^{2}H_2$]-hydrocinnamaldehyde (**Scheme S3**)

Reactions were conducted in 0.5 mL of ($^{2}H_5$)-Tris-^{2}HCl (100 mM, p^{2}H 8.0) containing 5 mM substrate, 2 vol.% [$^{2}H_6$]-DMSO, 0.5 mM NAD^{+}, and 500 μg of (*S*)- or (*R*)-ADH, or ene-reductase. The mixture was presaturated with H_2 gas prior to addition of the *Chemo-Bio* catalyst at a loading of 400 μg. The reactions were shaken at 500 rpm under a steady flow of H_2 for 16 hours. The ADH reactions were analyzed by chiral HPLC (**Fig. S 12**) and ^{1}H NMR spectroscopy (**Fig. S 13**), according to the procedures for similar reactions reported previously.[1] The ene reductase reactions were analyzed by GC (**Fig. S 14**) and ^{1}H NMR spectroscopy (**Fig. S 15**), according to the procedures for similar reactions reported previously.[1] The analysis revealed that the reactions proceeded to > 90% conversion, with only a single product being formed in each case, without the occurrence of side reactions which could plausibly occur at the platinum sites. The incorporation of ^{2}H at the target sites was also >95% in all cases.

Scheme S1

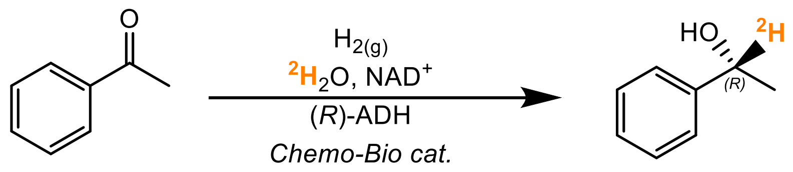

Scheme S2

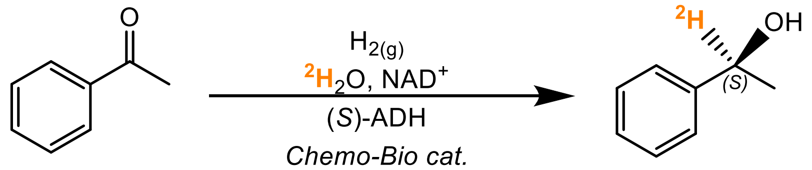

Scheme S3

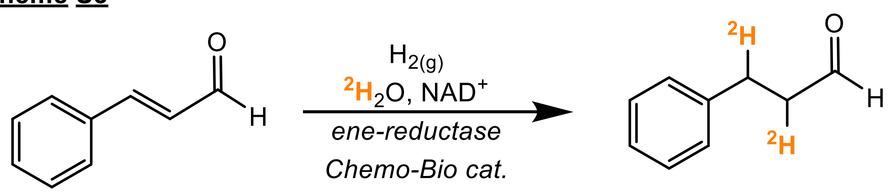

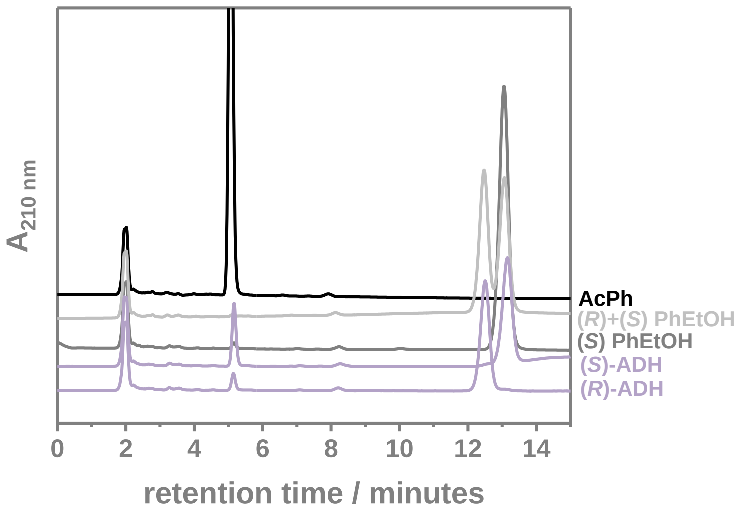

Fig. S 12 Chiral-HPLC analysis of the products of the H_2-driven reductive deuteration of AcPh by the *Chemo-Bio* system coupled to either the (*S*)- or (*R*)-ADH. Commercial standards of the substrate AcPh (black) and of racemic PhEtOH (light grey) and (*S*)-PhEtOH (dark grey) are shown as the upper traces. The lower traces show the reaction products, demonstrating the high conversion and perfect enantioselectivity of the *Chemo-Bio*/ADH system.

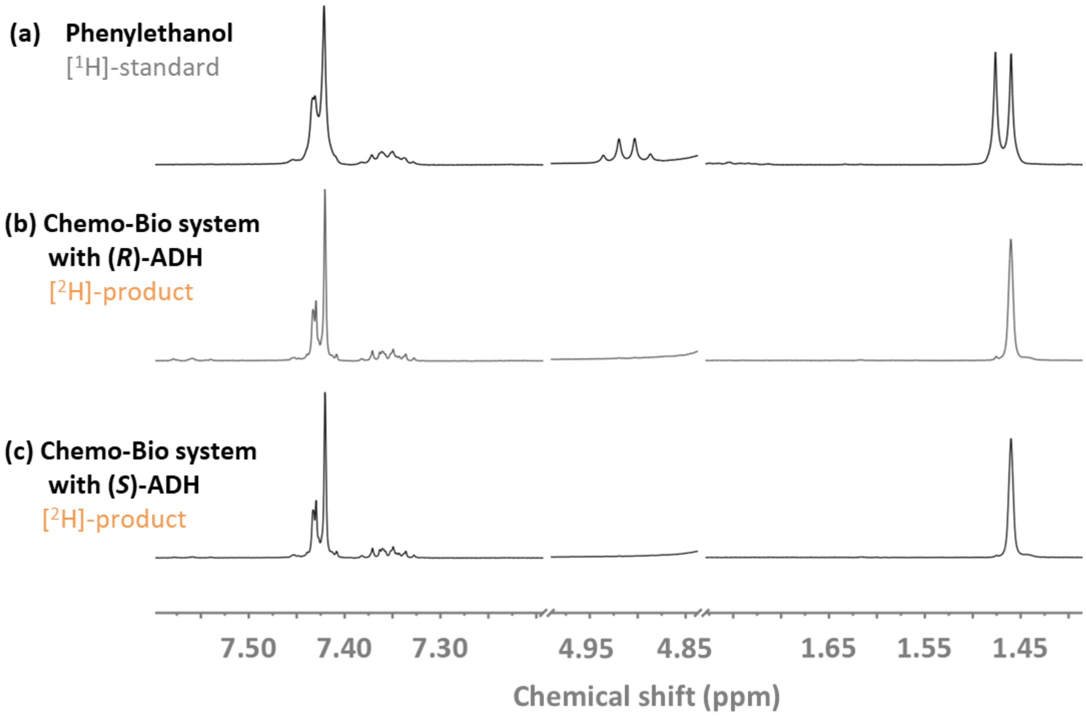

Fig. S 13 ^{1}H NMR spectroscopic analysis (^2H_2O, p^{2}H 8.0, 500 MHz, 293 K) of the H_2-driven reductive deuteration of AcPh by the *Chemo-Bio* system coupled to (*R*)- and (*S*)-ADH enzymes. **(a)** A commercial sample of unlabeled PhEtOH. **(b)** The reaction product from (*R*)-ADH. **(c)** The reaction product from (*S*)-ADH. The spectra demonstrate the >95% ^{2}H incorporation achieved by the *Chemo-Bio* system.

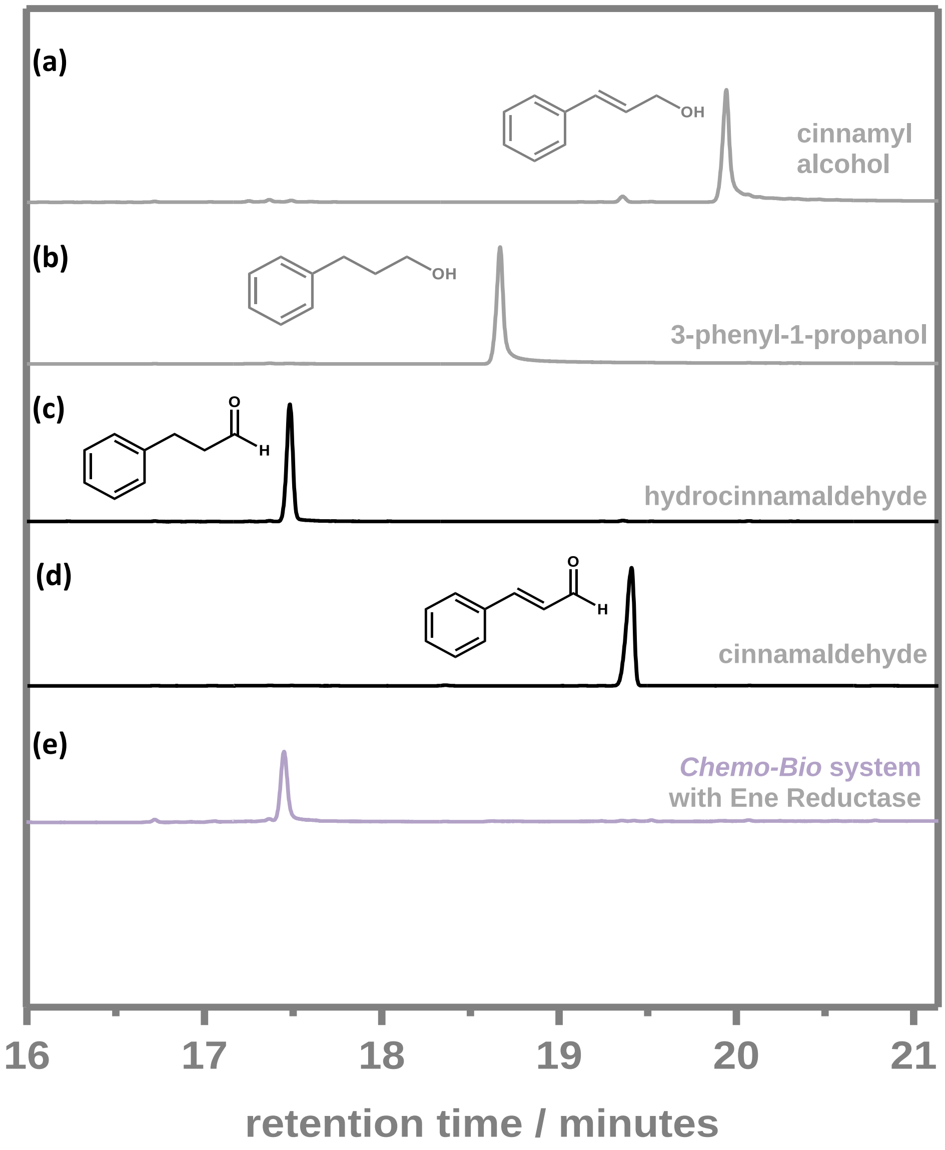

Fig. S 14 GC analysis of the product from the H_2-driven reductive deuteration of cinnamaldehyde by the *Chemo-Bio* system coupled to an ene-reductase. **(a)**, **(b)**, and **(c)** show potential products, and **(d)** shows the starting material; **(e)** shows the product from the reaction, and demonstrates the high conversion and chemoselectivity for hydrocinnamaldehyde.

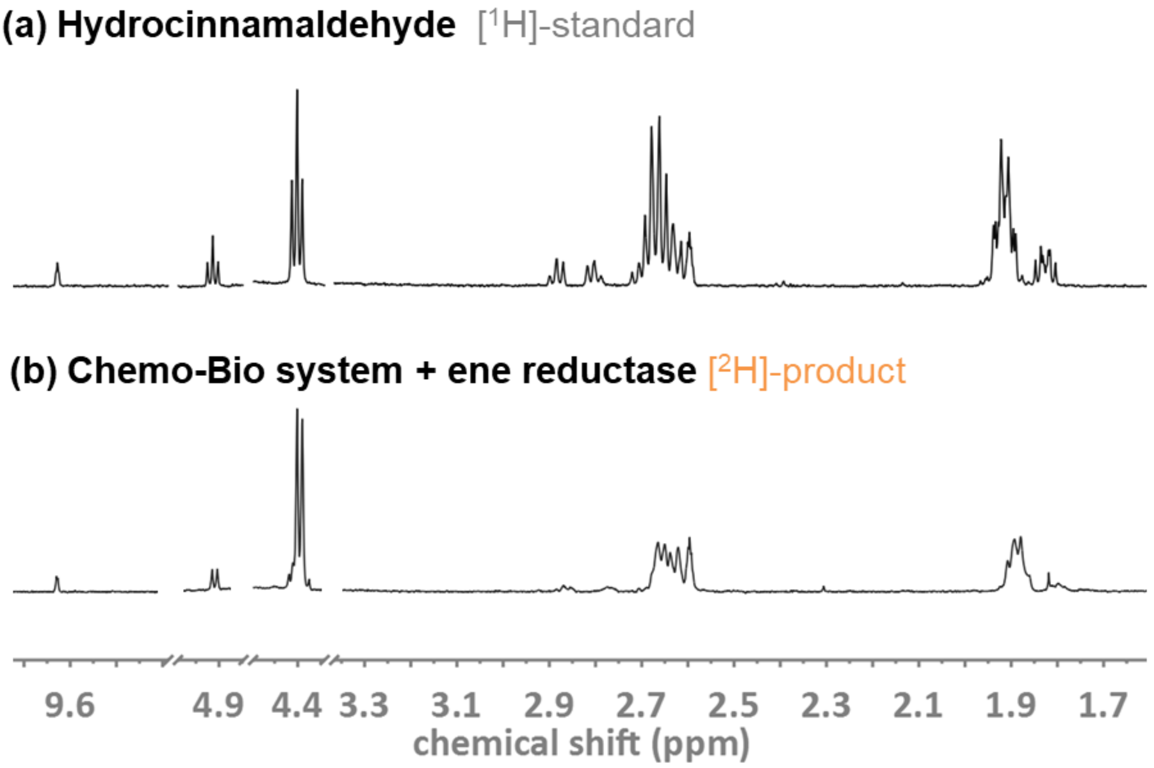

Fig. S 15 ^{1}H NMR spectroscopic analysis ($^{2}H_2O$, p^{2}H 8.0, 500 MHz, 293 K) of the H_2-driven reductive deuteration of cinnamaldehyde by the *Chemo-Bio* system coupled to an ene-reductase. **(a)** A commercial sample of unlablled hydrocinnamaldehyde. **(b)** The ene-reductase reaction product. The spectra demonstrate the >95% ^{2}H incorporation achieved by the *Chemo-Bio* system.

S.3 Supplementary References

(1) Rowbotham, J. S.; Ramirez, M. A.; Lenz, O.; Reeve, H. A.; Vincent, K. A. Bringing Biocatalytic Deuteration into the Toolbox of Asymmetric Isotopic Labelling Techniques. *Nat. Commun.* **2020**, *11* (1), 1454.

(2) Reeve, H. A.; Lauterbach, L.; Lenz, O.; Vincent, K. A. Enzyme-Modified Particles for Selective Biocatalytic Hydrogenation by Hydrogen-Driven NADH Recycling. *ChemCatChem* **2015**, *7* (21), 3480–3487.

(3) Lauterbach, L.; Lenz, O. Catalytic Production of Hydrogen Peroxide and Water by Oxygen-Tolerant [NiFe]-Hydrogenase during H_2 Cycling in the Presence of O_2. *J. Am. Chem. Soc.* **2013**, *135* (47), 17897–17905.

(4) Reeve, H. A.; Lauterbach, L.; Lenz, O.; Vincent, K. A. Enzyme-Modified Particles for Selective Biocatalytic Hydrogenation by Hydrogen-Driven NADH Recycling. *ChemCatChem* **2015**, *7* (21), 3480–3487.

(5) Lauterbach, L.; Idris, Z.; Vincent, K. A.; Lenz, O. Catalytic Properties of the Isolated Diaphorase Fragment of the NAD^+-Reducing [NiFe]-Hydrogenase from Ralstonia Eutropha. *PLoS One* **2011**, *6* (10), e25939.

(6) Evans, R. M.; Armstrong, F. A. Electrochemistry of Metalloproteins: Protein Film Electrochemistry for the Study of E. Coli [NiFe]-Hydrogenase-1. In *Methods in Molecular Biology (Clifton, N.J.)*; Fontecilla-Camps, J. C., Nicolet, Y., Eds.; Methods in Molecular Biology; Humana Press: Totowa, NJ, 2014; Vol. 1122, pp 73–94.

(7) Birrell, J. A.; Hirst, J. Investigation of NADH Binding, Hydride Transfer, and NAD^+ Dissociation during NADH Oxidation by Mitochondrial Complex I Using Modified Nicotinamide Nucleotides. *Biochemistry* **2013**, *52* (23), 4048–4055.

(8) Mostad, S. B.; Glasfeld, A. Using High Field NMR to Determine Dehydrogenase Stereospecificity with Respect to NADH. *J. Chem. Educ.* **1993**, *70* (6), 504–506.

(9) Wang, X.; Saba, T.; Yiu, H. H. P.; Howe, R. F.; Anderson, J. A.; Shi, J. Cofactor NAD(P)H Regeneration Inspired by Heterogeneous Pathways. *Chem* **2017**, *2* (5), 621–654.

(10) Jaegfeldt, H. A Study of the Products Formed in the Electrochemical Reduction of Nicotinamide-Adenine-Dinucleotide. *J. Electroanal. Chem. Interfacial Electrochem.* **1981**, *128*, 355–370.

(11) Ali, I.; Gill, A.; Omanovic, S. Direct Electrochemical Regeneration of the Enzymatic Cofactor 1,4-NADH Employing Nano-Patterned Glassy Carbon/Pt and Glassy Carbon/Ni Electrodes. *Chem. Eng. J.* **2012**, *188*, 173–180.

(12) Saba, T.; Burnett, J. W. H.; Li, J.; Kechagiopoulos, P. N.; Wang, X. A Facile Analytical Method for Reliable Selectivity Examination in Cofactor NADH Regeneration. *Chem. Commun.* **2020**, *56* (8), 1231–1234.

(13) Sazanov, L. A.; Hinchliffe, P. Structure of the Hydrophilic Domain of Respiratory Complex I from *Thermus Thermophilus*. *Science* **2006**, *311* (5766), 1430–1436.

(14) Berrisford, J. M.; Sazanov, L. A. Structural Basis for the Mechanism of Respiratory Complex I. *J. Biol. Chem.* **2009**, *284* (43), 29773–29783.

(15) Biasini, M.; Bienert, S.; Waterhouse, A.; Arnold, K.; Studer, G.; Schmidt, T.; Kiefer, F.; Cassarino, T. G.; Bertoni, M.; Bordoli, L.; Schwede, T. SWISS-MODEL: Modelling Protein Tertiary and Quaternary Structure Using Evolutionary Information. *Nucleic Acids Res.* **2014**, *42* (W1), W252–W258.
